# From traits to daily experiences: An adaptation of the Selflessness/Self-centeredness Inventory to day-level assessment

**DOI:** 10.1371/journal.pone.0351154

**Published:** 2026-06-23

**Authors:** Lucas David, Nicolas Pellerin, Michaël Dambrun

**Affiliations:** 1 Laboratoire de Psychologie Sociale et COgnitive, Université Clermont Auvergne, Clermont-Ferrand, France; 2 Activités Physiques et Sportives et processus PSYchologiques: recherche sur les Vulnérabilités, Université de Nîmes, Nîmes, France; 3 Laboratoire d’Ingénierie pour les Systèmes Complexes, Université Clermont Auvergne, Aubière, France; Akademia Jagiellońska w Toruniu: Akademia Jagiellonska, CZECHIA

## Abstract

Conceptualized as distinct psychological functionings respectively linked to authentic-durable and fluctuating happiness, selflessness and self-centeredness have recently been the subject of instrument development to evaluate their manifestations at the trait-level. Along the lines of this work, the goal of the current research was to adapt the psychometric instrument at the day-level: the Selflessness/Self-centeredness Inventory – Day-level (SSI-D). Consistent with the trait-version, both exploratory analysis (Study 1, *N* = 853) and confirmatory analysis (Study 2, *N* = 265) indicated that the SSI-D measured seven factors organized into two latent variables (i.e., selflessness divided into four components, self-centeredness divided into three dimensions). Correlational analysis (Study 3, *N* = 1409) provided initial evidence that day-level selflessness and self-centeredness can be differentiated psychometrically and showed theoretically meaningful patterns of association with mental health, interpersonal health, and pro-environmental outcomes. Invariance analyses were satisfactory, and preliminary validity evidence was generally consistent with theoretical expectations, although further validation using more comprehensive and jointly administered criterion measures is needed.

## Introduction

In the field of psychology of consciousness, numerous models have been created to describe altered states of consciousness. Taves [1] describes these states (including mystical experiences, undifferentiated unity, and self- or ego-dissolution) as nonordinary experiences which imply an alteration in the sense of self. The literature proposes a diversity of theoretical concepts. These concepts have their own particularities in terms of definition and manifestations, but they also share transversal dimensions. Among all these models, at the interface between social psychology, consciousness psychology, and positive psychology, the Self-centeredness/Selflessness Happiness Model (SSHM) [2] describes two complementary modes of psychological functioning: selflessness and self-centeredness. Selflessness and self-centeredness differ from other conceptualizations by operating at both the trait (i.e., a stable disposition, in the sense of a general psychological functioning extended in time) and the state (i.e., an immediate and transient experience that is limited in time) levels, as distinct and whole modes of psychological functioning characterized by multiple components [2–4]. State and trait influence themselves; the traits are reinforced by repeated experiences, while experiences derive from the activity of the trait components. Thus, selflessness and self-centeredness are conceived as overall ways of functioning present in everyone [2,5].

To define these two modes of psychological self-functioning, selflessness refers to a reduction in self-referential activity, including feelings, thoughts, and sensations centered on the self [3]. This reduced self-focus is accompanied by a broader sense of continuity and diminished differentiation between oneself and one’s physical and social environment, that is, a self-transcendent mode of functioning that appears across manifestations of selflessness [3]. In this respect, selflessness has been linked to the notion of unified consciousness, which similarly combines weakened self-environment boundaries with reduced identity-related self-processing [6]. However, selflessness should not be equated with adjacent concepts such as mindfulness or self-transcendence. Mindfulness primarily refers to an attentional mode of awareness [7,8], whereas self-transcendence, especially its relational dimension [9,10], refers more specifically to a transient experience involving altered self-boundaries and intense positive affect, such as bliss [11]. By contrast, selflessness is conceptualized in the SSHM as a broader mode of psychological functioning characterized by multiple components rather than a single attentional stance or transient altered experience [4]. Within this framework, selflessness is expected to foster emotional stability and a feeling of being in harmony, thereby contributing to authentic-durable happiness (i.e., a sustainable form of happiness characterized by tranquility and inner peace) [2].

By contrast, self-centeredness refers to a mode of functioning marked by heightened self-referential activity and a strong perceived distinction between oneself and the environment [3]. In this mode, thoughts, emotions, and sensations are experienced as highly self-relevant, and attention is absorbed by them, a process close to experiential fusion [12]. Thus, self-centeredness is not reducible to egocentric motives alone; rather, it reflects a broader organization of experience in which the self becomes the central frame of reference. This heightened self-salience favors hedonic approach-avoidance dynamics, such that positive stimuli are pursued and negative ones are avoided [13–15]. According to the SSHM, the experience of self-centeredness promotes both afflictive affects (e.g., jealousy, anger) and stimulus-driven pleasures, thereby fostering fluctuating happiness [2]. Here, “fluctuating” refers to the temporal lability of happiness, that is, the tendency for well-being and ill-being to alternate over time. More specifically, the hedonic approach-avoidance dynamic associated with self-centeredness makes happiness strongly dependent on whether experiences are construed as self-relevant gains or as self-relevant threats. Because these appraisals are inherently unstable and can change from one situation to the next, positive and negative affective states are more likely to alternate over time. In this way, hedonic reactivity does not merely influence the intensity of affect, but also its temporal instability, thereby contributing directly to fluctuating happiness [15].

Numerous studies have examined the SSHM framework across dispositional and experiential levels of analysis. At the dispositional level, because studies were mainly cross-sectional, happiness was assessed through subjective reports (i.e., individuals’ appraisal of happiness). Such studies demonstrated the links between selflessness, authentic-durable happiness and positive mental health, and the links between self-centeredness, fluctuating happiness and negative mental health [5,16–19]. At the experiential level, longitudinal experience-sampling studies made it possible to operationalize happiness more directly, by examining within-person variations in happiness across repeated assessments over time. Two experience-sampling studies confirmed that immediate and transient experiences of selflessness enhance happiness and prospectively stabilize it [20,21]. However, two important limitations of this work are the heterogeneity of instruments used to assess the modes of self-functioning (i.e., no specific, dedicated and validated tool), and the lack of studies on self-centeredness compared to selflessness [4].

To address these limitations, David et al. [4] recently developed the Selflessness/Self-centeredness Inventory – Trait (SSI-T) at the dispositional level. The SSI-T assesses selflessness through four components: connection with humanity, connection with nature, compassion, and decentering. The first two reflect a weakened distinction between self and environment, expressed as a universal sense of connection extending to other people and the natural world [4,19]. Compassion corresponds to the motivational component of selflessness and refers to a broad orientation toward harmony with others and the environment [2,4]. Decentering captures the reduction of self-referential activity through processes such as meta-awareness, disidentification, non-reactivity, acceptance, cognitive defusion, and self-as-context [11,22–24]. By contrast, the SSI-T assesses self-centeredness through three components: self-focus, self-importance, and hedonic process. Self-focus refers to immersion in one’s own sensations, thoughts, and emotions, in connection with processes such as experiential avoidance, cognitive fusion, and self-as-content [3,23]. Self-importance reflects the salience and centrality of the self, its needs, and its desires [4]. Finally, the hedonic process corresponds to the motivational dimension of self-centeredness, organized around approach toward pleasure and avoidance of displeasure [2,4,5]. Importantly, beyond its psychometric qualities, the validation of the SSI-T supported its incremental value by showing that it predicted mental health indicators above and beyond existing measures of related constructs, such as self-transcendence and ego-related functioning [4]. This suggests that selflessness and self-centeredness, as defined in the SSHM, are not fully captured by adjacent instruments and require a dedicated measure.

In the current research, we aim to extend David et al.’s [4] rationale to the day-level, to develop a reliable and valid psychometric instrument that would assess selflessness and self-centeredness as psychological functionings closer to experience. We made the methodological choice to focus on the day-level (i.e., “today”) for being distinct from global dispositions and getting closer to experience, in accordance with the day reconstruction method [25]. Such a framework is not the equivalent of pure immediate experiences but remains distinct from traits and restricted in time to illustrate a distinct level of functioning, intermediate between traits and momentary states. Hence, since the day-level scale evaluates components of psychological self-functioning, we expect that the same components would characterize selflessness and self-centeredness at both the overall dispositional level and the day-level. Thus, we hypothesize that the assessment of selflessness and self-centeredness at the day-level (i.e., measuring selflessness and self-centeredness in limited periods of time, involving less memory reconstruction and being closer to experience) would yield a two-latent-variable structure (i.e., the two modes of psychological functioning) composed of the seven statistically distinct factors identified by David et al. [4]: “connection with humanity”, “connection with nature”, “compassion”, and “decentering”, “self-focus”, “self-importance”, and “hedonic process”. Accordingly, we expected selflessness and self-centeredness to be differentiated at the day-level through distinct component profiles, consistent with previous trait-level findings [4,5,16].

In order to assess the construct validity of the scale, two categories of indicators were studied in the present research: (1) self-loss and dissolution of self-boundaries, and (2) mental health. Since the current research is cross-sectional, these indicators were assessed through subjective appraisals. Self-loss and dissolution of self-boundaries are dimensions closely related to all forms of nonordinary experiences, especially transcendence-like experiences [1,26]. By definition and given its components, these dimensions would be positively related to selflessness, especially dissolution of self-boundaries [3,6,20,21]. By contrast, self-centeredness, by reinforcing the sense of the self, would not be related to self-boundaries. Concerning the relation between self-centeredness and self-loss, predictions are more complex. On the one hand, as self-centeredness implies a stable structure of the self, it would not be related to self-loss. On the other hand, given the presence of self-focus and experiential fusion in self-loss experiences, positive relations with self-centeredness might be observed [4,10]. Regarding mental health, day-level selflessness would be expected to increase positive mental health, especially authentic-durable happiness [20,21], and decrease negative mental health [19]. In contrast, day-level self-centeredness would be accompanied by fluctuating happiness and decreasing mental health, notably due to the presence of ruminations and self-threat in negative mental health [16,19,27].

Additionally, with an exploratory view, two other categories of indicators were explored: relationship quality and relationship with nature (through ecoanxiety, ecological concerns and behaviors). This addition was made to examine relations with selflessness and self-centeredness beyond individual health, in line with recent research (see David et al. [4], and Stinus et al. [28] for an example of research on the effect of connectedness). It is predicted that selflessness would be positively related to relationship quality and relationship with nature, especially due to its connection and compassion components extended to the social and physical environment. These components would increase appreciation and concern for others and nature. Conversely, self-centeredness would have an overall null to negative relation to these indicators because of its focus on the self and its separation from the overall environment, leading to unstable appreciation and concern for others and nature, depending on their effect on the self.

## Study 1

The aim of the first study was to identify the factor structure of the Selflessness/Self-centeredness Inventory – Day-level (SSI-D) from a set of items measuring different aspects of selflessness and self-centeredness. This set of items was broadly the same as in the previous research on the trait-version [4].

### Method

#### Participants.

A sample of French participants was recruited on social media (Facebook, Messenger, Instagram) to complete the online questionnaire. The recruitment period ran from February 13, 2024, to February 25, 2024. Participants read and approved an electronic consent form prior to participating in the study. The research was presented as a study on daily experiences. Potential outliers were screened using standardized z-scores, with values greater than or equal to |3.00| were considered extreme. No participants met this criterion; therefore, no cases were excluded on this basis. The final sample comprised *N* = 853 participants (615 females; *M* = 51.1 years; *SD* = 12.3). In terms of educational level, the most represented diploma was the master’s degree or higher (*n* = 295). The most represented social class was the middle one (*n* = 596).

#### Material.

This study consisted of an online questionnaire developed on Qualtrics. The questionnaire included an information notice with a consent form, four socio-demographic questions (i.e., age, gender, level of diploma, social class), and measures for the scale development on selflessness and self-centeredness.

To assess selflessness and self-centeredness, we relied on the pool of items used in the pretest study of the trait-version [4]. Then, the general instruction of the trait-version (i.e., “We are interested in your experiences in your life in general. Below is a list of things people sometimes experience. Using the scale, please indicate for each statement how much you experience this in your life in general.”) was adapted to the day-level: “We are interested in your current experiences. Below is a list of things people sometimes experience. Using the scale, please indicate for each statement how much you experience this today.” Participants were asked to answer on a 7-point scale ranging from 1 (*Not at all*) to 7 (*Totally*). Consistent with Kahneman et al.’s [25] day reconstruction method, we used “today” as the level of analysis. It is important to recognize that “today” still involves a brief reconstruction and captures a mixture of momentary states and day-level experiences. Such daily reports are also not purely psychological, as affective and cognitive functioning are partly shaped by biological rhythms and preceding physiological states such as sleep duration/quality [25,29]. However, since “today” is a short and delimited period of time relatively easy to appraise from participants’ point of view with a reduced memory bias, that remains an acceptable approach for getting closer to experience while remaining distinct from the overall dispositional level. Items were adapted to this instruction and are presented below.

In line with David et al. [4], selflessness measures were grouped into three dimensions: connection (including both social and natural dimensions), decentering, and compassion.

**Connection:** We used four scales to assess connection: the Allo-Inclusive Identity scale (AII) [30], the Metapersonal Self scale (MPS) [31], the Shared Identity scale (adapted from Khan et al. [32]), and two dimensions of the Mystical Orientation scale (i.e., oneness and timelessness) [33]. The AII scale measured allo-inclusivity through two dimensions, connection with others and connection with nature. Among the 16 original items, eight were used. Four items assessed connection with others (e.g., “Today, I feel a strong connection between myself and the person I feel closest to.”) and four items assessed connection with the natural environment (e.g., “Today, I feel a strong connection between myself and a tree.”). The reliability of this scale was satisfactory (*ω* = 0.85). The MPS scale was measured with 10 items self-representations which refer to an essence beyond the individual and others, to a universal focus [31]. Among the original items, five were used (e.g., “Today, I feel a real sense of kinship with all living things.”). The reliability of this scale was satisfactory (*ω* = 0.72). The shared identity scale measured the perception of a common essence between participants and a reference group (here, humanity). This scale comprised five items (e.g., “Today, I have a feeling of unity with others.”). The reliability of this scale was satisfactory (*ω* = 0.89). Last, the two dimensions of the MOS measured the consciousness of the oneness of everything and the sense of timelessness, respectively [33]. Each dimension was assessed by three items (e.g., “Today, I sense the unity in all things”, “Today, I am conscious only of timelessness and eternity”). The reliability of the oneness dimension was satisfactory (*ω* = 0.85), but the reliability of the timelessness dimension was unsatisfactory (*ω* = 0.47).

**Decentering:** To assess decentering, we used the Metacognitive Processes of Decentering Scale (MPDS) [22] and the psychological flexibility dimension of the Multidimensional Psychological Flexibility Inventory (MPFI-24) [34]. The MPDS assessed three different decentering processes with 15 items in its original version. The French translation was provided by De Oliveira et al. [35]. For each process, the three most understandable items were selected (e.g., “Today, I am able to watch myself thinking.”). The first dimension measured meta-awareness, the second dimension measured disidentification, and the third dimension measured non-reactivity. The reliability of this scale was satisfactory (*ω* = 0.79). The psychological flexibility dimension of the MPFI-24 comprised 12 original items. Among them, six items were used: two items on acceptance, two items on the self-as-context and two items on cognitive defusion (e.g., “Today, I try to make peace with my negative thoughts and emotions rather than resisting them.”). The reliability of this factor was satisfactory (*ω* = 0.88).

**Compassion:** We used five scales to assess compassion: the Compassionate Goals scale [36], the dimensions of empathetic concern and perspective taking from the Interpersonal Reactivity Index (IRI) [37], the self-transcendence dimension of the Portrait Value Questionnaire (PVQ) [38], and the dimensions of kindness and common humanity from the Compassion Scale (CS) [39]. The Compassionate Goals scale included seven items to assess the focus on people’s well-being or inclusion. All seven items of this scale were used (e.g., “Today, I want to support others.”). The reliability of this scale was satisfactory (*ω* = 0.75). The empathetic concern dimension of the IRI evaluated the emotional dimension of empathy. Three items assessing this dimension were used (e.g., “Today, I feel protective towards people being taken advantage of.”). The French translation was published by Gilet et al. [40]. The reliability of this subscale was unsatisfactory (*ω* = 0.57). The perspective taking dimension of the IRI evaluated the cognitive dimension of empathy. Three items assessing this dimension were used (e.g., “Today, I feel able to imagine how I would feel if I were in someone else’s place.”). The reliability of this subscale was unsatisfactory (*ω* = 0.47). The self-transcendence dimension of the PVQ measured benevolence and universalism values. The five items of this dimension were used (e.g., “Today, I want to be loyal to my friends, I want to devote myself to people close to me.”). The reliability of this scale was satisfactory (*ω* = 0.63). The kindness dimension of the CS assessed caring toward and concern for others with three items (e.g., “Today, my heart goes out to people who are unhappy.”). The common humanity dimension evaluated the recognition that everyone has an experience of suffering, with three items (e.g., “Today, I feel that suffering is just a part of the common human experience.”). The reliability of the kindness dimension was satisfactory (*ω* = 0.74), but the reliability of the common humanity dimension was unsatisfactory (*ω* = 0.53).

Still in line with David et al. [4], self-centeredness measures were grouped into three dimensions: self-focus, self-importance, and hedonic process.

**Self-focus:** We used three scales to assess self-focus: the rumination dimension of the Rumination-Reflection Questionnaire (RRQ) [41], the psychological inflexibility dimension of the Multidimensional Psychological Flexibility Inventory (MPFI-24) [34], and the personal distress dimension of the IRI. The rumination dimension of the RRQ comprised 12 items in its original version. Among the original items, five were used in this study (e.g., “Today, I ruminate or dwell over things that happen to me.”). The reliability of this scale was satisfactory (*ω* = 0.85). The MPFI-24 [34] included 12 items to assess psychological inflexibility processes. Among them, six were used: two items on experiential avoidance, two items on the self-as-content and two items on cognitive fusion (e.g., “Today, I try to distract myself when I feel unpleasant emotions.”). The reliability of the scale was satisfactory (*ω* = 0.83). Last, one item assessing the personal distress dimension of the IRI [37] was used (i.e., “Today, I could lose my self-control during emergencies.”). It was the only selected item because others’ formulations were close to anxiety, which could interfere with our results.

**Self-importance:** We used three scales to assess self-importance: the Egocentrism Scale [42], the narcissism dimension of the Dirty Dozen Dark Triad (DDDT) [43], and the self-enhancement dimension of the PVQ and the Self-Image Goals scale [36]. The Egocentrism Scale assessed egocentrism with 10 items. Among these original items, five were used in this study (e.g., “Today, I behave in a somewhat selfish way.”). The reliability of this scale was satisfactory (*ω* = 0.66). The narcissism dimension of the DDDT, translated into French by Savard et al. [44], comprised four items. All items were used (e.g., “Today, I want others to pay attention to me.”). The reliability of this scale was satisfactory (*ω* = 0.76). The self-enhancement dimension of the PVQ comprised four items to assess achievement and power values. All were used in this study (e.g., “Today, I want to be successful, I would like to impress other people.”). The reliability of this dimension was satisfactory (*ω* = 0.74). Last, the Self-Image Goals scale included six items to assess the focus on personal well-being or self-image. The six items of this scale were used (e.g., “Today, I want others to recognize or acknowledge my positive qualities.”). The reliability of this scale was satisfactory (*ω* = 0.70).

**Hedonic process:** To assess hedonic process, we used the Material Values Scale (MVS) [45] and the Behavioral Activation System (BAS) scale [46]. The MVS measured materialism in six items used in this study (e.g., “Today, I admire people who own expensive homes, cars, and clothes.”). These items were divided into three constructs: success, centrality, and happiness. The reliability of this scale was satisfactory (*ω* = 0.79). The BAS scale measured behavioral activation with 13 original items. Six items were used in this study. Among these items, two measured each of the following dimensions: reward responsiveness (e.g., “Today, I keep at it when I’m doing well at something.”), drive (e.g., “Today, I go out of my way to get things I want.”) and fun seeking (e.g., “Today, I crave excitement and new sensations.”). The reliability of this scale was satisfactory (*ω* = 0.67).

#### Procedure.

Participation in this study was anonymous. Respondents individually self-administered the questionnaire. The median completion duration was about 19 minutes. After certifying their consent, participants were exposed to the general instructions and asked to complete the block of items on psychological functioning. These items were presented in random order, across all scales, and spread over four pages. Finally, participants completed the socio-demographic questions. Data were collected using Qualtrics software. All procedures performed in this study were in accordance with the Declaration of Helsinki, reviewed and approved by the Université Clermont Auvergne’s ethics committee (IRB00011540-2022-93). Participants read and approved an electronic consent form prior to participating in the study. The data were analyzed anonymously.

#### Data analysis.

Statistical analyses were performed using Jamovi software (Version 2.7.24). An exploratory factor analysis was carried out to identify the factor structure. The Shapiro-Wilk test suggested a violation of normality on the entire data set. No data transformation was applied prior to the analyses. This decision was made because no outliers were identified using the predefined |z| ≥ 3.00 criterion, and the non-normality did not appear to be attributable to a specific or consistent distributional pattern across variables. In particular, deviations were not systematically associated with marked skewness or kurtosis, and some variables identified as non-normal nevertheless remained within commonly accepted bounds on these indicators. The analysis was therefore carried out using the principal axis extraction method and the oblimin rotation. The number of factors was estimated with parallel analysis, which has better statistical properties than the eigenvalue-based criteria [47]. Next, items were introduced into exploratory structural equation modeling (ESEM) to determine if the seven factors could be grouped into a hierarchical structure of two second-order latent variables. Two models were computed: an ESEM configured with seven first-order factors (Model 1) and a higher-order ESEM where the seven first-order factors were grouped into two second-order latent variables (i.e., Model 2). These analyses were configured with the robust maximum likelihood method and the oblimin rotation. Both models were compared as a preliminary exploration of the hierarchical structure of the scale. The adequacy of the models to the data was determined on the basis of the following reference values: CFI > 0.90 (ideally 0.95), TLI > 0.90 (ideally 0.95), SRMR and RMSEA < 0.08 (ideally 0.05) [48,49]. Reference fits to compare models were: Δ_CFI_ > 0.01, Δ_TLI_ > 0.01, Δ_RMSEA_ > 0.015, Δ_SRMR_ > 0.030 and Δ_χ²_ tests (*p* < .05) [50–52]. Given the well-documented sensitivity of the chi-square statistic to sample size and non-normality [53], greater emphasis was placed on approximate fit indices (i.e., CFI, TLI, RMSEA, and SRMR) when interpreting model fit. Selflessness and self-centeredness scores were then calculated by averaging the corresponding items.

### Results

Overall, the scores of the 106 items were introduced into the exploratory factor analysis. With *N* = 853 participants and 106 variables, the ratio of participants per dimension was approximately 8:1. Bartlett’s test of sphericity was significant, *χ²* (5565) = 44897; *p* < .001. The Kaiser-Meyer-Olkin (KMO) test of sampling adequacy was satisfactory (*MSA* = 0.933). Measures of model fit were satisfactory. The Root Mean Square Error of Approximation (RMSEA) was 0.03. The model test was significant, *χ²* (4550) = 8293; *p* < .001. This factor analysis revealed a ten-factor solution (Factor 1: eigenvalue = 16.29; Factor 2: eigenvalue = 9.31; Factor 3: eigenvalue = 5.60; Factor 4: eigenvalue = 4.27; Factor 5: eigenvalue = 2.28; Factor 6: eigenvalue = 1.43; Factor 7: eigenvalue = 1.11; Factor 8: eigenvalue = 1.03; Factor 9: eigenvalue = 0.81; Factor 10: eigenvalue = 0.61). These factors explained 43.12% of the total variance.

With a view to selecting only the most discriminating items to obtain a balanced version of each dimension, items with a factor load of less than 0.50 on their primary factor and/or greater than 0.20 on a secondary factor were removed (for a similar methodology, see Hanley et al. [22]). Of the initial 106 items, 35 were retained. Following this selection, the analysis led to a seven-factor solution explaining 55.97% of the total variance. Factor 1 “self-focus” (eigenvalue = 6.70; % variance = 9.41) included three rumination items and two psychological inflexibility items. Factor 2 “connection with humanity” (eigenvalue = 4.10; % of variance = 9.32) grouped the five shared identity items. Factor 3 “connection with nature” (eigenvalue = 2.25; % variance = 8.60) included three allo-inclusivity items related to nature, one oneness item and one metapersonal self item. Factor 4 “compassion” (eigenvalue = 1.59; % variance = 8.41) grouped two compassionate goals items, two benevolence value items, and one kindness item. Factor 5 “self-importance” (eigenvalue = 1.07; % of variance = 7.87) grouped two narcissism items, two achievement value items, and one self-image goal item. Factor 6 “hedonic process” (eigenvalue = 0.64; % variance = 6.82) comprised four materialism items and one power value item. Factor 7 “decentering” (eigenvalue = 0.56; % variance = 5.54) grouped together four decentering process items and one psychological flexibility item. The selected items for each scale factor and their factor loadings are presented in Table 1. The internal reliability of each factor was satisfactory: *ω*_F1_ = 0.89; *ω*_F2_ = 0.89; *ω*_F3_ = 0.88; *ω*_F4_ = 0.87; *ω*_F5_ = 0.85; *ω*_F6_ = 0.82; and *ω*_F7_ = 0.76.

**Table 1:** Selected items for the Selflessness/Self-centeredness Inventory – Day-level following exploratory factor analysis (Study 1).

Factor	Coding	Description	Factor loading
Factor 1 – Self-focus			
	MPFI12	Today, I have distressing thoughts spinning on a loop in my head. *Aujourd’hui, j’ai des pensées pénibles qui tournent en boucle dans ma tête.*	0.863
	RUM4	Today, I am affected by negative thoughts and emotions. *Aujourd’hui, je suis habité(e) par mes pensées et mes émotions négatives.*	0.836
	RUM1	Today, I ruminate or dwell over things that happen to me. *Aujourd’hui, je rumine ou pense sans m’arrêter à des choses qui me sont arrivées.*	0.809
	MPFI11	Today, I keep going back to what happened during a past argument or disagreement. *Aujourd’hui, je suis focalisé(e) sur ce qui s’est produit lors d’un désagrément passé.*	0.758
	RUM3	Today, I rehash in my mind recent things I’ve said or done. *Aujourd’hui, je remanie dans mon esprit des choses récentes que j’ai pu dire ou faire.*	0.611
Factor 2 – Connection with humanity			
	SID5	Today, I think of human beings as part of a single group. *Aujourd’hui, je pense que tous les êtres humains font partie d’un seul groupe.*	0.854
	SID1	Today, I think of human beings as part of one large family. *Aujourd’hui, je pense que les êtres humains font partie d’une même grande famille.*	0.829
	SID2	Today, I have a sense of “we-ness” with all human beings. *Aujourd’hui, j’ai le sentiment d’être « nous » avec tous les êtres humains.*	0.762
	SID3	Today, I have a feeling of unity with others. *Aujourd’hui, j’éprouve un sentiment d’unité avec tous les êtres humains.*	0.713
	SID4	Today, I share the same identity with all human beings. *Aujourd’hui, je partage la même identité que tous les êtres humains.*	0.656
Factor 3 – Connection with nature			
	AII7	Today, I feel a strong connection between myself and the Earth. *Aujourd’hui, je sens une forte connexion entre moi et la terre.*	0.823
	AII4	Today, I feel a strong connection between myself and a tree. *Aujourd’hui, je sens une forte connexion entre moi et un arbre.*	0.809
	AII8	Today, I feel a strong connection between myself and an eagle soaring in the sky. *Aujourd’hui, je sens une forte connexion entre moi et un aigle qui plane dans le ciel.*	0.800
	MOS2	Today, I feel at one with the universe. *Aujourd’hui, je me sens en union avec l’univers.*	0.644
	MPS5	Today, I experience myself as extending into everything else. *Aujourd’hui, je me vois comme une extension de tout le reste.*	0.520
Factor 4 – Compassion			
	CPG1	Today, I want to support others. *Aujourd’hui, je souhaite soutenir les autres.*	0.812
	STV3	Today, I want to help people around me and care for them. *Aujourd’hui, je veux aider les gens autour de moi et prendre soin des autres.*	0.809
	CS1	Today, I want to be there for others in times of difficulty. *Aujourd’hui, je souhaite être là pour les autres dans les moments difficiles.*	0.791
	CPG4	Today, I want to make a positive difference in someone else’s life. *Aujourd’hui, je souhaite apporter une contribution positive à la vie de quelqu’un d’autre.*	0.711
	STV4	Today, I want to be loyal and to devote myself to people close to me. *Aujourd’hui, je veux être loyal(e) et me dévouer pour mon entourage.*	0.633
Factor 5 – Self-importance			
	SEV2	Today, I want to show my abilities, I want people to admire what I do. *Aujourd’hui, je souhaite montrer mes capacités et que les gens admirent ce que je fais.*	0.808
	NAR1	Today, I want others to admire me. *Aujourd’hui, je recherche l’admiration des autres.*	0.801
	SEV3	Today, I want to be successful, I would like to impress other people. *Aujourd’hui, je veux réussir et impressionner les autres.*	0.672
	SIG1	Today, I want to get others to recognize or acknowledge my positive qualities. *Aujourd’hui, je souhaite amener les autres à reconnaître ou à admettre mes qualités positives.*	0.657
	NAR2	Today, I want others to pay attention to me. *Aujourd’hui, je veux que les autres m’accordent de l’attention.*	0.595
Factor 6 – Hedonic process			
	SEV1	Today, I want to be rich, to have a lot of money and expensive things. *Aujourd’hui, je veux être riche, avoir beaucoup d’argent et des choses qui valent très cher.*	0.822
	MVS4	Today, I like to have a lot of luxury in my life. *Aujourd’hui, j’aime avoir beaucoup de luxe dans ma vie.*	0.692
	MVS6	Today, I imagine my life better if I owned some things I don’t have. *Aujourd’hui, je m’imagine être plus heureux/se si je me permettais d’acheter plus de choses.*	0.634
	MVS5	Today, I think my life would be better if I owned certain things I don’t have. *Aujourd’hui, je pense que ma vie serait meilleure si je possédais certaines choses que je n’ai pas.*	0.569
	MVS1	Today, I admire people who own expensive homes, cars, and clothes. *Aujourd’hui, j’admire les gens qui possèdent des maisons, des voitures et des vêtements coûteux.*	0.547
Factor 7 – Decentering			
	MPDS2	Today, I am aware of unpleasant thoughts or feelings without immediately reacting to them. *Aujourd’hui, je suis conscient(e) des pensées ou des sentiments désagréables sans y réagir immédiatement.*	0.660
	MPDS5	Today, I am able to notice distressing thoughts or feelings without reacting. *Aujourd’hui, je suis capable de remarquer mes pensées ou mes sentiments pénibles sans réagir.*	0.636
	MPDS4	Today, I watch my thoughts and emotions come and go like clouds. *Aujourd’hui, je regarde mes pensées et mes émotions aller et venir comme des nuages.*	0.560
	MPDS6	Today, I am able to watch my thoughts and feelings like someone watching a movie. *Aujourd’hui, je suis capable d’observer mes pensées et mes sentiments comme quelqu’un qui regarde un film.*	0.559
	MPFI1	Today, I am open to observing unpleasant thoughts and feelings without interfering with them. *Aujourd’hui, je suis ouvert(e) à observer les pensées et les émotions désagréables sans interférer avec celles-ci.*	0.541

MPFI: Multidimensional Psychological Flexibility Inventory, RUM: rumination – Rumination-Reflection Questionnaire, SID: Shared Identity scale, AII: Allo-Inclusive Identity scale, MOS: Mystical Orientation scale, MPS: Metapersonal Self scale, CPG: Compassionate Goals scale, STV: self-transcendent values – Portrait Value Questionnaire, CS: Compassion Scale, SEV: self-enhancement values – Portrait Value Questionnaire, NAR: narcissism – Dirty Dozen Dark Triad, MVS: Material Values Scale, MPDS: Metacognitive Processes of Decentering Scale.

To evaluate the hierarchical structure of the scale, the 35 selected items were introduced into two ESEM models. Model 1, configured with seven first-order factors, depicted satisfactory fits: χ²(371) = 808.5, *p* < .001; SRMR = 0.017; RMSEA = 0.037; 95% CI RMSEA [0.031, 0.039]; CFI = 0.970; TLI = 0.952; AIC = 99044; BIC = 100440. All items significantly and strongly loaded into their respective factor (β > 0.50, *p* < .001). Cross-loading values were systematically lower than |β| = 0.20, indicating limited cross-loading influences, consistent with the prior EFA selection. Model 2, configured with seven first-order factors grouped into two second-order latent variables, depicted satisfactory fits: χ²(363) = 808.5, *p* < .001; SRMR = 0.017; RMSEA = 0.038; 95% CI RMSEA [0.034, 0.041]; CFI = 0.970; TLI = 0.950; AIC = 99060; BIC = 100494. All items significantly and strongly loaded into their respective factor (β > 0.50, *p* < .001). Cross-loading values were still systematically lower than |β| = 0.20. Models 1 and 2 were essentially equivalent according to conventional criteria (Δ_*χ²*_ = 0.00; Δ_SRMR_ = 0.000; Δ_RMSEA_ = 0.001; Δ_CFI_ = 0.000; Δ_TLI_ = 0.002; Δ_AIC_ = 16; Δ_BIC_ = 54), indicating that they cannot be distinguished on empirical fit grounds. Model 2 was preferred because it was more theoretically grounded by the assumptions of the SSHM [2] and previous findings [4,5,16]. On this basis, a selflessness score and a self-centeredness score were calculated by averaging the items of their respective factors. These two scores were weakly negatively correlated: *ρ*(851) = −.197; *p* < .001.

### Discussion

The aim of this study was to develop selflessness and self-centeredness day-level measures by selecting items from a large set of measures. As a reminder, this day-level operationalization should be understood as referring to experiences reported for “today”, involving brief retrospective reconstruction, rather than to strict momentary states assessed through experience sampling protocols. Seven factors (i.e., “self-focus”, “self-importance”, “hedonic process”, “connection with humanity”, “connection with nature”, “compassion”, and “decentering”) organized under two latent variables (i.e., “self-centeredness” and “selflessness”) were identified by our analyses, combining exploratory factor analysis and higher-order ESEM comparison. This structure was consistent with our hypotheses and with the trait structure [4]. Moreover, by comparing the 35 items of this day-level version to the 32 items of the trait-version, 25 items are common to both scales. As the item selection for the day-level version has been made with an exploratory method, which supports the stability of results.

One limitation of the study is that four out of 21 dimensions used to build the initial item pool showed low internal consistency (i.e., ω < 0.60). This may have introduced some construct-irrelevant variance into the exploratory factor analysis. However, these dimensions were not used as substantive composite scores in the analyses; they served as provisional item pools in an item-selection procedure (as the main purpose of the analysis was to identify the most discriminating items for the scale development). In addition, these low-reliability dimensions (i.e., timelessness, empathetic concern, perspective taking, and common humanity) represented a minority of the initial pool and none of their items were retained in the final factor structure, indicating that any construct-irrelevant variance associated with these dimensions did not carry over into the retained scale.

## Study 2

The second study aimed to confirm the factor structure of the SSI-D using a confirmatory method on an independent sample. The structure of the SSI-D identified in Study 1 was compared to alternative structures with structural equation models. To avoid relying on a sample too similar to that of Study 1, and to reduce the risk of overlap between participants recruited from the same general population source, Study 2 was conducted with a distinct student sample. Although more homogeneous than the Study 1 sample, this design provided a useful cross-validation of the factor structure in a different population. Moreover, given the frequent use of student participants in psychological research [54,55], it was important to include a student sample alongside a larger general-population sample. This allowed us to examine whether the scale performs adequately in a population commonly studied in psychology.

### Method

#### Participants.

Participants were French students from the Université Clermont Auvergne, invited to complete the online questionnaire. The recruitment period ran from March 13, 2024, to April 06, 2024. Participants read and approved an electronic consent form prior to participating in the study. The research was presented as a study on daily experiences. Potential outliers were screened using standardized z-scores (i.e., values greater than or equal to |3.00| considered extreme). No participants met this criterion; therefore, no cases were excluded on this basis. The final sample comprised *N* = 265 participants (180 females; *M* = 21.3 years; *SD* = 4.8). Regarding educational level, the most represented year of study was the third-year bachelor’s degree (*n* = 106). The most represented social class was the middle one (*n* = 175).

#### Material and procedure.

This study consisted of an online questionnaire developed on Qualtrics. The questionnaire included the 35 items selected from Study 1 (see Table 1), presented in a random order. The instructions for these items were identical to those used in the previous study. Socio-demographic questions appeared at the end of the questionnaire.

Participation in this study was anonymous. Respondents individually self-administered the questionnaire after certifying their consent. The median completion duration was about 9 minutes. Ethical approval, consent, and anonymized data processing were identical to Study 1.

#### Data analysis.

Statistical analyses were performed using Jamovi software (Version 2.7.24). The statistical method in Study 2 consisted of a confirmatory approach using the structural equation modeling framework provided by the semlj package of Jamovi. Given the absence of normality, analyses were performed using the robust maximum likelihood method. No transformation was applied, as no outliers were identified and the deviation from normality did not follow a consistent pattern across variables. Different structural equation models were tested to determine which factor structure presented the best fit to the data. Five models were compared. They varied according to their number of first-order (i.e., latent variables directly measured by items) and second-order (i.e., latent variables formed by groups of first-order variables) variables. Model 1 was a single-factor structure comprising all 35 items; Model 2 was a two-factor structure (i.e., selflessness and self-centeredness as two first-order variables); Model 3 was a seven-factor structure (i.e., seven first-order variables corresponding to the seven hypothesized dimensions); Model 4 was a hierarchical seven-factor structure (i.e., seven first-order variables grouped into a second-order variable); Model 5 was the hypothesized factor structure (seven first-order variables grouped into two second-order variables: selflessness and self-centeredness).

The following reference values from Browne and Cudeck [48] and Hu and Bentler [49] were used as thresholds to determine the models’ adequacy to the data: CFI > 0.90 (ideally 0.95), TLI > 0.90 (ideally 0.95), SRMR and RMSEA < 0.08 (ideally 0.05). To compare models, reference values were: Δ_CFI_ > 0.01, Δ_TLI_ > 0.01, Δ_RMSEA_ > 0.015, Δ_SRMR_ > 0.030, and Δ_χ²_ tests (*p* < .05) [50–52].

### Results

Scores of the 35 items were introduced into structural equation modeling. The ratio of participants per variable was approximately 7.6:1. As shown in Table 2, only models 3 and 5 presented acceptable fit indices. Furthermore, the fits of models 3 and 5 were essentially equivalent according to conventional criteria (Δ_*χ²*_ = 52, *p* < .001; Δ_CFI_ = 0.009; Δ_TLI_ = 0.007; Δ_SRMR_ = 0.008; Δ_RMSEA_ = 0.002; Δ_AIC_ = 26; Δ_BIC_ = 21), indicating that they cannot be clearly distinguished on empirical fit alone.

**Table 2:** Adequacy indices for the tested structural equation models (Study 2).

Model	Chi-square	Degree of freedom	CFI	TLI	SRMR	RMSEA	95% CI RMSEA	AIC	BIC
Model 1	4049	560	0.249	0.193	0.172	0.152	[0.147, 0.157]	35593	35969
Model 2	3126	559	0.451	0.416	0.130	0.130	[0.125, 0.135]	34672	35052
Model 3	978	539	0.919	0.910	0.060	0.051	[0.045, 0.057]	32565	33016
Model 4	1109	553	0.893	0.885	0.102	0.058	[0.052, 0.064]	32668	33068
Model 5	1030	552	0.910	0.903	0.068	0.053	[0.047, 0.059]	32591	32995

Model 1: 1 factor (35 items), Model 2: 1 factor “Selflessness” and 1 factor “Self-centeredness”, Model 3: 7 factors without higher-ordered variable, Model 4: Model 3 with 1 higher-ordered variable, Model 5: Model 3 with 2 higher-ordered variables (i.e., “Selflessness” and “Self-centeredness”).

Since covariations between the first-order factors were modeled in Model 5 but not in Model 3, Model 5 was the most parsimonious. In addition, Model 5 was more theoretically grounded by the assumptions of the SSHM [2] and previous findings obtained for trait assessments [4,5,16]. Accordingly, Model 5 was retained and was interpreted as supporting the hierarchical model describing seven first-order factors organized into two second-order factors (i.e., “selflessness” and “self-centeredness”). On this basis, we computed selflessness and self-centeredness scores by averaging their respective items. These scores were uncorrelated (*r*(263) = 0.09; *p* = .144). In addition, all items had significant factor loadings on their respective factor (cf. Table 3). Finally, each factor had a satisfactory internal reliability (*ω*_F1_ = 0.89; *ω*_F2_ = 0.86; *ω*_F3_ = 0.86; *ω*_F4_ = 0.89; *ω*_F5_ = 0.87; *ω*_F6_ = 0.85; and *ω*_F7_ = 0.78).

**Table 3:** Standardized factor loadings of validated Selflessness/Self-centeredness Inventory – Day-level items in the selected structural equation model (Study 2).

Factor	Coding	Description	β
Factor 1 – Self-focus			
	MPFI12	Today, I have distressing thoughts spinning on a loop in my head. *Aujourd’hui, j’ai des pensées pénibles qui tournent en boucle dans ma tête.*	0.881 ^a^
	RUM1	Today, I ruminate or dwell over things that happen to me. *Aujourd’hui, je rumine ou pense sans m’arrêter à des choses qui me sont arrivées.*	0.822 ^a^
	MPFI11	Today, I keep going back to what happened during a past argument or disagreement. *Aujourd’hui, je suis focalisé(e) sur ce qui s’est produit lors d’un désagrément passé.*	0.807 ^a^
	RUM4	Today, I am affected by negative thoughts and emotions. *Aujourd’hui, je suis habité(e) par mes pensées et mes émotions négatives.*	0.757 ^a^
	RUM3	Today, I rehash in my mind recent things I’ve said or done. *Aujourd’hui, je remanie dans mon esprit des choses récentes que j’ai pu dire ou faire.*	0.648 ^a^
Factor 2 – Connection with humanity			
	SID2	Today, I have a sense of “we-ness” with all human beings. *Aujourd’hui, j’ai le sentiment d’être « nous » avec tous les êtres humains.*	0.837 ^a^
	SID5	Today, I think of human beings as part of a single group. *Aujourd’hui, je pense que tous les êtres humains font partie d’un seul groupe.*	0.759 ^a^
	SID3	Today, I have a feeling of unity with others. *Aujourd’hui, j’éprouve un sentiment d’unité avec tous les êtres humains.*	0.751 ^a^
	SID1	Today, I think of human beings as part of one large family. *Aujourd’hui, je pense que les êtres humains font partie d’une même grande famille.*	0.729 ^a^
	SID4	Today, I share the same identity with all human beings. *Aujourd’hui, je partage la même identité que tous les êtres humains.*	0.617 ^a^
Factor 3 – Connection with nature			
	AII7	Today, I feel a strong connection between myself and the Earth. *Aujourd’hui, je sens une forte connexion entre moi et la terre.*	0.856 ^a^
	AII4	Today, I feel a strong connection between myself and a tree. *Aujourd’hui, je sens une forte connexion entre moi et un arbre.*	0.810 ^a^
	AII8	Today, I feel a strong connection between myself and an eagle soaring in the sky. *Aujourd’hui, je sens une forte connexion entre moi et un aigle qui plane dans le ciel.*	0.723 ^a^
	MOS2	Today, I feel at one with the universe. *Aujourd’hui, je me sens en union avec l’univers.*	0.723 ^a^
	MPS5	Today, I experience myself as extending into everything else. *Aujourd’hui, je me vois comme une extension de tout le reste.*	0.574 ^a^
Factor 4 – Compassion			
	STV3	Today, I want to help people around me and care for them. *Aujourd’hui, je veux aider les gens autour de moi et prendre soin des autres.*	0.866 ^a^
	CPG1	Today, I want to support others. *Aujourd’hui, je souhaite soutenir les autres.*	0.841 ^a^
	CS1	Today, I want to be there for others in times of difficulty. *Aujourd’hui, je souhaite être là pour les autres dans les moments difficiles.*	0.786 ^a^
	CPG4	Today, I want to make a positive difference in someone else’s life. *Aujourd’hui, je souhaite apporter une contribution positive à la vie de quelqu’un d’autre.*	0.770 ^a^
	STV4	Today, I want to be loyal and to devote myself to people close to me. *Aujourd’hui, je veux être loyal(e) et me dévouer pour mon entourage.*	0.675 ^a^
Factor 5 – Self-importance			
	SEV2	Today, I want to show my abilities, I want people to admire what I do. *Aujourd’hui, je souhaite montrer mes capacités et que les gens admirent ce que je fais.*	0.803 ^a^
	NAR1	Today, I want others to admire me. *Aujourd’hui, je recherche l’admiration des autres.*	0.799 ^a^
	SEV3	Today, I want to be successful, I would like to impress other people. *Aujourd’hui, je veux réussir et impressionner les autres.*	0.773 ^a^
	SIG1	Today, I want to get others to recognize or acknowledge my positive qualities. *Aujourd’hui, je souhaite amener les autres à reconnaître ou à admettre mes qualités positives.*	0.723 ^a^
	NAR2	Today, I want others to pay attention to me. *Aujourd’hui, je veux que les autres m’accordent de l’attention.*	0.664 ^a^
Factor 6 – Hedonic process			
	SEV1	Today, I want to be rich, to have a lot of money and expensive things. *Aujourd’hui, je veux être riche, avoir beaucoup d’argent et des choses qui valent très cher.*	0.824 ^a^
	MVS1	Today, I admire people who own expensive homes, cars, and clothes. *Aujourd’hui, j’admire les gens qui possèdent des maisons, des voitures et des vêtements coûteux.*	0.749 ^a^
	MVS4	Today, I like to have a lot of luxury in my life. *Aujourd’hui, j’aime avoir beaucoup de luxe dans ma vie.*	0.738 ^a^
	MVS5	Today, I think my life would be better if I owned certain things I don’t have. *Aujourd’hui, je pense que ma vie serait meilleure si je possédais certaines choses que je n’ai pas.*	0.682 ^a^
	MVS6	Today, I imagine my life better if I owned some things I don’t have. *Aujourd’hui, je m’imagine être plus heureux/se si je me permettais d’acheter plus de choses.*	0.660 ^a^
Factor 7 – Decentering			
	MPFI1	Today, I am open to observing unpleasant thoughts and feelings without interfering with them. *Aujourd’hui, je suis ouvert(e) à observer les pensées et les émotions désagréables sans interférer avec celles-ci.*	0.692 ^a^
	MPDS5	Today, I am able to notice distressing thoughts or feelings without reacting. *Aujourd’hui, je suis capable de remarquer mes pensées ou mes sentiments pénibles sans réagir.*	0.674 ^a^
	MPDS6	Today, I am able to watch my thoughts and feelings like someone watching a movie. *Aujourd’hui, je suis capable d’observer mes pensées et mes sentiments comme quelqu’un qui regarde un film.*	0.667 ^a^
	MPDS2	Today, I am aware of unpleasant thoughts or feelings without immediately reacting to them. *Aujourd’hui, je suis conscient(e) des pensées ou des sentiments désagréables sans y réagir immédiatement.*	0.627 ^a^
	MPDS4	Today, I watch my thoughts and emotions come and go like clouds. *Aujourd’hui, je regarde mes pensées et mes émotions aller et venir comme des nuages.*	0.567 ^a^

MPFI: Multidimensional Psychological Flexibility Inventory, RUM: rumination – Rumination-Reflection Questionnaire, SID: Shared Identity scale, AII: Allo-Inclusive Identity scale, MOS: Mystical Orientation scale, MPS: Metapersonal Self scale, CPG: Compassionate Goals scale, STV: self-transcendent values – Portrait Value Questionnaire, CS: Compassion Scale, SEV: self-enhancement values – Portrait Value Questionnaire, NAR: narcissism – Dirty Dozen Dark Triad, MVS: Material Values Scale, MPDS: Metacognitive Processes of Decentering Scale.

^a^*p* < .001.

It is important to note that both Model 3 and Model 5 fits were acceptable, but not strictly satisfactory. Accordingly, the hierarchical structure should be interpreted as a useful psychometrically acceptable summary of the seven first-order dimensions, supported by acceptable but not optimal fit, and requiring further independent replication. Interestingly, their fits appear significantly lower than those obtained for the two ESEM models tested in Study 1, which were the same models. Indeed, Study 1’s Model 1 tested the same seven-factor structure as Study 2’s Model 3, while Study 1’s Model 2 tested the same hierarchical structure as Study 2’s Model 5. The gap between fits may reflect constraints applied in the confirmatory approach, in which cross-loading values are fixed to zero, unlike in ESEM. For this reason, two additional ESEM models were estimated in this study, in order to determine if cross-loadings contribute in part to misfit. The models were performed using the robust maximum likelihood method. Model 3b was configured with seven first-order factors and Model 5b was configured with the seven first-order factors grouped into two second-order latent variables. Model 3b depicted acceptable fits: χ²(371) = 625.7, *p* < .001; SRMR = 0.026; RMSEA = 0.051; 95% CI RMSEA [0.044, 0.058]; CFI = 0.945; TLI = 0.912; AIC = 32548; BIC = 33601. Only one item presented a cross-loading value superior to |β| = 0.20. This item, from the ‘connection with humanity’ dimension, cross-loaded to the ‘connection with nature’ dimension (β = 0.347), which is theoretically meaningful as both dimensions assess forms of connectedness. Model 5b also depicted acceptable fits: χ²(363) = 625.7, *p* < .001; SRMR = 0.026; RMSEA = 0.052; 95% CI RMSEA [0.045, 0.059]; CFI = 0.943; TLI = 0.907; AIC = 32564; BIC = 33645. Cross-loading showed the same pattern as in Model 3b. Both ESEM models were equivalent according to reference criteria (Δ_*χ²*_ = 0.00; Δ_SRMR_ = 0.000; Δ_RMSEA_ = 0.001; Δ_CFI_ = 0.002; Δ_TLI_ = 0.005; Δ_AIC_ = 16; Δ_BIC_ = 44). Interestingly, Model 3b differed from Model 3 in some indicators (Δ_*χ²*_ = 352.3, *p* < .001; Δ_SRMR_ = −0.034; Δ_RMSEA_ = 0.000; Δ_CFI_ = 0.026; Δ_TLI_ = 0.002; Δ_AIC_ = −17; Δ_BIC_ = 585), as was the case for Model 5b and Model 5 (Δ_*χ²*_ = 404.3, *p* < .001; Δ_SRMR_ = −0.042; Δ_RMSEA_ = −0.001; Δ_CFI_ = 0.033; Δ_TLI_ = 0.004; Δ_AIC_ = −27; Δ_BIC_ = 650). ESEM models depicted lower SRMR and higher CFI values, while BIC increases indicated more complexity. Thus, a part of the misfit in confirmatory analyses can be attributed to cross-loadings, but improvements were not sufficient to reach strictly satisfactory thresholds.

### Discussion

This study aimed to evaluate the adequacy of the SSI-D factor structure obtained in Study 1 compared with alternative factor structures. Although the Study 2 sample was more restricted than that of Study 1, its use served a cross-validation purpose: confirming the structure in a distinct population, commonly studied in psychological research and recruited from a different source, rather than reproducing the analysis in a highly similar general-population sample with possible participant overlap. This choice strengthens the independence of the confirmatory step, although it also implies reduced heterogeneity and therefore calls for caution regarding generalizability. Besides, both samples were French, which may limit cross-cultural generalizability.

Overall, the results were broadly consistent with the exploratory structure and with the expected hierarchical model: a first latent variable, selflessness, was represented by connection with humanity, connection with nature, compassion, and decentering; while a second latent variable, self-centeredness, was represented by self-focus, self-importance, and hedonic process. However, model fit remained acceptable rather than fully satisfactory, which calls for a cautious interpretation of the higher-order structure. The additional ESEM analyses suggested that part of the misfit may be attributable to the restrictive zero cross-loading constraints imposed by SEM models, since ESEM showed somewhat improved fit. At the same time, these improvements were not sufficient or generalized enough to yield clearly satisfactory fit, indicating that cross-loadings alone do not fully account for the remaining misfit. Importantly, as in Study 1, the first-order and higher-order ESEM models were essentially equivalent, just as the corresponding SEM models were, so that the data did not allow a clear empirical preference between the two representations. The retention of the hierarchical model should therefore be understood primarily as theory-driven and parsimonious rather than as unequivocally superior on statistical grounds alone. This choice was guided by the SSHM framework, in which selflessness and self-centeredness are conceptualized as broad modes of psychological functioning expressed through distinct components, and by previous preliminary findings supporting a comparable organization at the trait level [4,5,16]. It also has the applied advantage of allowing future research to examine both overall day-level self-functioning scores and specific component scores, depending on the research question. These results remained consistent with the SSI-T structure [4], while suggesting that the precise hierarchical organization would benefit from further replication and testing across samples and designs. Thus, at this stage, the results provide preliminary support for distinguishing day-level selflessness and self-centeredness as two broader functionings characterized by specific factors, in line with expectations.

## Study 3

The aim of the third study was to investigate the construct validity of the SSI-D by examining its relationships with the dissolution of self-boundaries and happiness measures. In addition, relationship quality and relationship with nature outcomes have been added to explore their associations with selflessness and self-centeredness at this level of analysis, and to determine if relations are consistent with the overall trait-level [4].

### Method

#### Participants.

A sample of French participants was recruited on social media (Facebook, Messenger, Instagram) to complete the online questionnaire. The recruitment period ran from April 08, 2024, to April 23, 2024. Participants read and approved an electronic consent form prior to participating in the study. Again, the research was presented as a study on daily experiences. Potential extreme cases were examined using standardized z-scores (|z| ≥ 3.00). No outliers were identified, so all participants were retained. The final sample comprised *N* = 1409 participants (974 females; *M* = 47.8 years; *SD* = 11.7). Regarding educational level, the most represented diploma was the bachelor’s degree (*n* = 359), followed by the master’s degree or higher (*n* = 352). The most represented social class was the middle one (*n* = 863).

#### Material.

This study consisted of an online questionnaire developed on Qualtrics. The questionnaire included an information notice with a consent form, four socio-demographic questions (i.e., age, gender, level of diploma, social class), the SSI-D, and measures for construct validity and exploratory correlations.

To assess divergent validity, we examined the associations between the SSI-D, social desirability, and socio-demographic variables (i.e., gender, educational level, social class), as these variables are often used in this way in similar research.

**Social desirability:** Social desirability was assessed by the French self-deception and other-deception measure developed by Tournois et al. [56]. This scale comprised two dimensions: self-deception (i.e., trickery toward oneself, giving oneself a positive image) and other-deception (i.e., trickery toward other people, giving them a positive image of ourselves). In this study, the 10 items that presented the highest factor loading were used, comprising five items for self-deception (e.g., “I am always optimistic.”) and five items for other-deception (e.g., “I always respect the law.”). Participants were asked to indicate the extent to which each item applied to them, on a Likert scale ranging from 1 (*Totally false*) to 7 (*Totally true*). The reliability of both dimensions was satisfactory (respectively *ω* = 0.82 and *ω* = 0.60).

To assess convergent validity, we examined the associations between the SSI-D and two categories of variables: a) self-loss, self-boundaries, and mindfulness as related constructs, and b) mental health and happiness, as related phenomena. All measures were adapted to reflect “today’s” experiences.

**Self-loss and dissolution of self-boundaries:** Dimensions of self-loss and dissolution of self-boundaries were assessed by the measures developed by Canby et al. [26]. These measures evaluated the 14 following dimensions: unity with all things (*ω* = 0.85), boundary diminishment with others (*ω* = 0.81), boundary diminishment with objects (*ω* = 0.92), self-loss (*ω* = 0.89), loss of ownership of thoughts and feelings (*ω* = 0.87), loss of agency (*ω* = 0.83), non-specific self-loss (*ω* = 0.94), voluntary surrender (*ω* = 0.87), spiritual insight (*ω* = 0.80), power and confidence (*ω* = 0.79), loss of time and space (*ω* = 0.85), ineffability (*ω* = 0.78), positive affect (*ω* = 0.80), and negative affect (*ω* = 0.92). Participants were invited to answer on a Likert scale ranging from 1 (Not at all or don’t understand) to 7 (Totally).

**Perceived body boundaries salience:** The salience of the perceived body boundaries was measured by the single PBBS item [3]. This item was a visual analog scale ranging from 0 (*My body boundaries are almost imperceptible*) to 100 (*My body boundaries are extremely salient*). The item was accompanied by a figure representing seven human bodies whose body boundaries ranged from almost imperceptible (on the left pole) to extremely prominent (on the right pole).

**Mindfulness:** Experience of mindfulness was evaluated with the Toronto Mindfulness Scale (TMS) [57]. This scale comprised 13 items divided into two dimensions: curiosity (e.g., “Today, I am curious about my reactions to things.”), and decentering (e.g., “Today, I experience myself as separate from my changing thoughts and feelings.”). Participants were asked to answer on a Likert scale ranging from 1 (*Not at all*) to 7 (*Very much*). The reliability of both dimensions was satisfactory (respectively *ω* = 0.90 and *ω* = 0.78).

**Positive mental health:** To assess positive mental health, participants reported their level of life satisfaction by answering the following item: “Today, I am satisfied with my life.” on a visual analog scale ranging from 1 (*Strongly disagree*) to 100 (*Strongly agree*). The use of visual analog scales in this aim was previously realized by Cuchet et al. [58,59]. Participants also indicated their level of positive emotions on a similar visual analog scale ranging from 1 (*Very low*) to 100 (*Very high*). In addition, two items from the Subjective Authentic-Durable Happiness Scale (SADHS) [14] were added (e.g., “Today, what is your level of inner calm?”). Participants rated their level of authentic-durable happiness on a visual analog scale from 1 (*Very low*) to 100 (*Very high*). The reliability of this measure was satisfactory (*ω* = 0.76).

**Negative mental health:** Still in line with Cuchet et al. [58,59], to assess negative mental health, participants rated their level of anxiety, depression, and negative emotions on three visual analog scales from 1 (*Very low*) to 100 (*Very high*). Anxiety and depression items have been previously validated against the Hospital Anxiety and Depression scale, a standard clinical measure commonly used in France [60]. In addition, two items from the Subjective Fluctuating Happiness Scale (SFHS) [14] were added (e.g., “Today, my level of serenity is very changeable.”). Participants answered on a visual analog scale from 1 (*Strongly disagree*) to 100 (*Strongly agree*). The reliability of this measure was satisfactory (*ω* = 0.80).

Complementary to mental health measures, indicators of relationship quality and relationship with nature have been added to test broader predictions of the SSHM.

**Relationship quality:** The relationship quality was evaluated by the dimensions adapted from Snijders et al. [61]: trust in honesty (e.g., “People around me are honest about my problems.”), trust in benevolence (e.g., “People around me are concerned about my welfare.”), satisfaction (e.g., “I am happy with my relationships with the people around me.”), affective commitment (e.g., “I feel emotionally attached to people around me.”), and affective conflict (e.g., “I am angry with people around me.”). In this study, participants responded according to their relationships with those around them, by indicating their level of agreement for the 15 items on a Likert scale ranging from 1 (*Strongly disagree*) to 7 (*Strongly agree*). The reliability of each dimension was satisfactory (respectively *ω* = 0.85, *ω* = 0.85, *ω* = 0.93, *ω* = 0.84 and *ω* = 0.85).

**Relationship with nature:** The relationship with nature was assessed by three indicators: ecoanxiety [62], ecological concern (i.e., recognition of the impacts of ecological issues), and intention to behave in an eco-friendly way (adapted from Clayton & Karazsia [63]). The Hogg Ecoanxiety Scale (HEAS) [62] comprised 13 items to evaluate four dimensions of ecoanxiety. In this study, only three dimensions were used: 4 items for affective symptoms (e.g., “Today, I feel nervous, anxious, or on edge.”), 3 items for rumination (e.g., “Today, I am unable to stop thinking about losses to the environment.”), and 3 items for anxiety about personal impact (e.g., “Today, I feel anxious about the impact of my personal behaviors on the earth.”). The dimension of behavioral symptoms was not included because its items were irrelevant for “today’s” experience. The reliability of the scale was satisfactory (*ω* = 0.90). Ecological concern and behavioral intentions were adaptations of dimensions from the Climate Change Anxiety scale [63]. Indeed, the three experience items and four behavior items were reworded to refer to ecological issues in general, not only climate change. Participants answered on a Likert scale ranging from 1 (*Strongly disagree*) to 7 (*Strongly agree*). Reliability of both dimensions was satisfactory (respectively *ω* = 0.74 and *ω* = 0.82).

#### Procedure.

Participation in this study was anonymous. Respondents individually self-administered the questionnaire. After certifying their consent, participants were exposed to the SSI-D and one-third of the remaining measures presented in a random order. Indeed, we did not present all measures to avoid creating an excessively long and exhausting questionnaire. The first subsample (*n* = 358) completed the PBBS, self-loss, and dissolution of self-boundaries measures, the second subsample (*n* = 421) completed social desirability and mindfulness measures, and the subsample (*n* = 408) completed mental health, relationship quality and relationship with nature measures. The median completion duration was about 12 minutes. Finally, participants completed the socio-demographic questions. Data were collected using Qualtrics software. Ethical approval, consent, and anonymized data processing were identical to Study 1.

#### Data analysis.

Statistical analyses were performed using Jamovi software (Version 2.7.24). The reliability of these scales was assessed using McDonald’s omega index. The Shapiro-Wilk test suggested an overall violation of normality. No transformation was applied, as no outliers were identified and the deviation from normality did not follow a consistent pattern across variables. Correlational analyses were therefore carried out using Spearman’s *ρ*. Relationships between the SSI-D and socio-demographic variables were examined in multiple linear regressions. Importantly, all correlations with selflessness in this study were also tested with the control of self-centeredness, leading to the same results. The results were also the same with self-centeredness when controlling selflessness.

### Results

#### Divergent validity.

**Social desirability:** Both selflessness and self-centeredness were uncorrelated with other-deception (respectively *ρ*(419) = −.005; *p* = .927 and *ρ*(419) = −.083; *p* = .087). Selflessness correlated positively with self-deception (*ρ*(419) =.324; *p* < .001) while self-centeredness correlated negatively with it (*ρ*(419) = −.480; *p* < .001).

**Socio-demographic variables:** Both selflessness and self-centeredness were uncorrelated with gender (respectively *p* = .104; *β* = −0.05; 95% CI [−0.11, 0.01] and *p* = .290; *β* = −0.03; 95% CI [−0.08, 0.03]), educational level (respectively *p* = .224; *β* = 0.04; 95% CI [−0.02, 0.10] and *p* = .159; *β* = −0.04; 95% CI [−0.10, 0.02]), and social class (respectively *p* = .207; *β* = 0.04; 95% CI [−0.02, 0.10] and *p* = .158; *β* = −0.04; 95% CI [−0.10, 0.02]).

#### Convergent validity.

**Age:** Selflessness correlated positively with age (*p* < .001; *β* = 0.11; 95% CI [0.05, 0.17]) while self-centeredness correlated negatively with age (*p* < .001; *β* = −0.29; 95% CI [−0.35, −0.24]).

**Self-loss and dissolution of self-boundaries:** As shown in Table 4 (below the diagonal), among the 14 analyzed dimensions, selflessness correlated with 10 dimensions (i.e., unity with all things, boundary diminishment with others, boundary diminishment with objects, loss of agency, voluntary surrender, spiritual insight, power and confidence, loss of time and space, and both positive and negative affect). Self-centeredness correlated with 8 dimensions (i.e., self-loss, loss of ownership of thoughts and feelings, loss of agency, non-specific self-loss, power and confidence, loss of time and space, ineffability, and negative affect). Given the important correlations between these dimensions and well-being (see correlations with both positive and negative affect), partial correlations were investigated by controlling positive and negative affect (see Table 4, above the diagonal). Then, selflessness correlated positively with 9 dimensions (i.e., unity with all things: *ρ*(356) =.611, *p* < .001; boundary diminishment with others: *ρ*(356) =.451, *p* < .001; boundary diminishment with objects: *ρ*(356) =.353, *p* < .001; self-loss: *ρ*(356) =.128, *p* = .015; voluntary surrender: *ρ*(356) =.268, *p* < .001; spiritual insight: *ρ*(356) =.331, *p* < .001; power and confidence: *ρ*(356) =.229, *p* < .001; loss of time and space: *ρ*(356) =.258, *p* < .001; and ineffability: *ρ*(356) =.146, *p* = .006) while self-centeredness correlated with 2 dimensions (i.e., positively with loss of agency: *ρ*(356) =.152, *p* = .004, and negatively with power and confidence: *ρ*(356) = −.108, *p* = .042).

**Table 4:** Raw correlations between selflessness, self-centeredness, and self-loss and dissolution of self-boundaries dimensions (Study 3).

	1	2	3	4	5	6	7	8	9	10	11	12	13	14	15	16
1	/	0.105^a^	0.611^c^	0.451^c^	0.353^c^	0.128 ^a^	0.058	−0.016	−0.004	0.268^c^	0.331^c^	0.229^c^	0.258^c^	0.146 ^b^	/	/
2	−0.083^b^	/	0.068	0.056	0.022	0.102	0.054	0.152 ^b^	0.042	−0.042	−0.045	−0.108 ^a^	−0.017	−0.060	/	/
3	0.662^c^	−0.025	/	0.639^c^	0.479^c^	0.276^c^	0.176^c^	0.145 ^b^	0.102	0.349^c^	0.453^c^	0.322^c^	0.248^c^	0.247^c^	/	/
4	0.476^c^	0.101	0.665^c^	/	0.592^c^	0.475^c^	0.338^c^	0.258^c^	0.349^c^	0.344^c^	0.439^c^	0.290^c^	0.281^c^	0.290^c^	/	/
5	0.365^c^	0.078	0.502^c^	0.632^c^	/	0.388^c^	0.246^c^	0.185^c^	0.219^c^	0.314^c^	0.353^c^	0.238^c^	0.287^c^	0.210^c^	/	/
6	0.012	0.402^c^	0.190^c^	0.484^c^	0.407^c^	/	0.568^c^	0.477^c^	0.626^c^	0.253^c^	0.199^c^	0.052	0.281^c^	0.434^c^	/	/
7	−0.059	0.372^c^	0.096	0.364^c^	0.288^c^	0.738^c^	/	0.481^c^	0.619^c^	0.204^c^	0.079	−0.002	0.239^c^	0.202^c^	/	/
8	−0.145 ^b^	0.484^c^	0.038	0.287^c^	0.232^c^	0.710^c^	0.708^c^	/	0.489^c^	0.268^c^	0.115 ^a^	0.018	0.333^c^	0.284^c^	/	/
9	−0.049	0.307^c^	0.096	0.415^c^	0.291^c^	0.753^c^	0.740^c^	0.666^c^	/	0.253^c^	0.096	0.006	0.231^c^	0.300^c^	/	/
10	0.329^c^	0.021	0.427^c^	0.461^c^	0.390^c^	0.324^c^	0.262^c^	0.285^c^	0.338^c^	/	0.532^c^	0.300^c^	0.315^c^	0.263^c^	/	/
11	0.397^c^	0.013	0.527^c^	0.553^c^	0.429^c^	0.290^c^	0.174^c^	0.187^c^	0.230^c^	0.631^c^	/	0.573^c^	0.360^c^	0.314^c^	/	/
12	0.333^c^	−0.155 ^b^	0.416^c^	0.369^c^	0.286^c^	0.035	−0.029	−0.038	0.024	0.383^c^	0.620^c^	/	0.235^c^	0.173 ^b^	/	/
13	0.179^c^	0.212^c^	0.224^c^	0.355^c^	0.343^c^	0.481^c^	0.440^c^	0.517^c^	0.422^c^	0.379^c^	0.414^c^	0.215^c^	/	0.405^c^	/	/
14	0.083	0.259^c^	0.227^c^	0.401^c^	0.304^c^	0.655^c^	0.495^c^	0.573^c^	0.543^c^	0.380^c^	0.427^c^	0.176^c^	0.566^c^	/	/	/
15	0.338^c^	−0.072	0.368^c^	0.384^c^	0.240^c^	0.141 ^b^	0.067	0.029	0.176^c^	0.403^c^	0.482^c^	0.356^c^	0.156 ^b^	0.281^c^	/	/
16	−0.190^c^	0.551^c^	−0.080	0.169 ^b^	0.148 ^b^	0.632^c^	0.619^c^	0.722^c^	0.530^c^	0.159 ^b^	0.158 ^b^	−0.073	0.430^c^	0.581^c^	0.030	/

Below the diagonal: raw correlations. Above the diagonal: partial correlations when controlling positive and negative affects. 1: selflessness, 2: self-centeredness, 3: unity with all things, 4: boundary diminishment with others, 5: boundary diminishment with objects, 6: self-loss, 7: loss of ownership of thoughts and feelings, 8: loss of agency, 9: non-specific self-loss, 10: voluntary surrender, 11: spiritual insight, 12: power and confidence, 13: loss of time and space, 14: ineffability, 15: positive affect, 16: negative affect.

^a^*p* < .05.

^b^*p* < .01.

^c^*p* < .001.

**Perceived body boundaries salience:** Selflessness correlated negatively with PBBS, *ρ*(356) = −.277; *p* < .001, while self-centeredness was uncorrelated with this variable: *ρ*(356) =.080; *p* = .130.

**Mindfulness:** Selflessness correlated positively with both curiosity (*ρ*(419) =.456; *p* < .001) and decentering (*ρ*(419) =.547; *p* < .001). Self-centeredness was uncorrelated with curiosity (*ρ*(419) =.026; *p* = .597) but correlated negatively with decentering (*ρ*(419) = −.185; *p* < .001).

**Positive mental health:** Selflessness correlated positively with life satisfaction (*ρ*(406) =.171; *p* < .001), positive emotions (*ρ*(406) =.282; *p* < .001), and authentic-durable happiness (*ρ*(406) =.259; *p* < .001), while self-centeredness correlated negatively with these three variables (respectively *ρ*(406) = −.420; *p* < .001; *ρ*(406) = −.425; *p* < .001; and *ρ*(406) = −.462; *p* < .001).

**Negative mental health:** Selflessness correlated negatively with negative emotions (*ρ*(406) = −.193; *p* < .001), anxiety (*ρ*(406) = −.162; *p* = .001), depression (*ρ*(406) = −.167; *p* < .001), and fluctuating happiness (*ρ*(406) = −.144; *p* = .001), while self-centeredness correlated positively with these four variables (respectively *ρ*(406) =.571; *p* < .001; *ρ*(406) =.514; *p* < .001; *ρ*(406) =.522; *p* < .001; and *ρ*(406) =.413; *p* < .001).

#### Correlations with exploratory indicators.

**Relationship quality:** Selflessness correlated positively with trust in honesty (*ρ*(406) =.101; *p* = .042), trust in benevolence (*ρ*(406) =.181; *p* < .001), satisfaction (*ρ*(406) =.139; *p* = .005), affective commitment (*ρ*(406) =.161; *p* = .001), and negatively with affective conflict (*ρ*(406) = −.117; *p* = .018). Self-centeredness correlated negatively with trust in honesty (*ρ*(406) = −.207; *p* < .001), trust in benevolence (*ρ*(406) = −.243; *p* < .001), satisfaction (*ρ*(406) = −.367; *p* < .001), affective commitment (*ρ*(406) = −.258; *p* < .001), and positively with affective conflict (*ρ*(406) =.426; *p* < .001).

**Relationship with nature:** Selflessness correlated positively with ecological concerns (*ρ*(392) =.283; *p* < .001), the intention to behave in an eco-friendly way (*ρ*(392) =.291; *p* < .001). No correlation was found between selflessness and ecoanxiety (*ρ*(392) =.086; *p* = .088). Self-centeredness correlated positively with ecoanxiety (*ρ*(392) =.352; *p* < .001) and negatively with ecological concerns (*ρ*(392) = −.168; *p* < .001) and intention to behave in an eco-friendly way (*ρ*(392) = −.234; *p* < .001).

### Discussion

The aim of this third study was to evaluate the construct validity of the scale and to explore its associations with various categories of outcomes (i.e., mental health, relationship quality, and relationship with nature).

Regarding convergent validity, positive associations between selflessness, self-loss and dissolution of self-boundaries dimensions, and mindfulness were in line with hypothesis. Indeed, at the day-level, selflessness is close to relational transcendence and to mindful experience [2,3,6,20,21]. Weakly negative to no associations with self-centeredness were also in line with predictions, as self-centeredness is characterized by a strong sense of self separated from its environment [2,3]. Lastly, correlations with mental health were also consistent with the theoretical background and previous studies [3,20,21]. It is important to underline the heterogeneity of the correlation sizes observed between the scale and the validity indicators. Indeed, if these results depict significant relations that are consistent with expectations regarding the validity of the scale, effect sizes indicate only partial overlap between variables. Then, daily levels of selflessness and self-centeredness are linked to but distinct from mental health, mindfulness, and self-loss and dissolution of self-boundaries dimensions. This is consistent with the view of measuring components of specific modes of psychological functioning.

Analyses concerning divergent validity showed no associations between the scale, other-deception, and three relevant socio-demographic variables, which were consistent with predictions. However, correlations involving self-deception should be interpreted with caution. On the one hand, this finding raises the possibility that, to some extent, high selflessness scores may capture a positively valued self-view, including a form of self-enhancement in the moral domain. In other words, participants reporting high selflessness may partly describe themselves in ways that are consistent with socially desirable or idealized self-perceptions. However, this interpretation does not seem sufficient to reduce the construct to a mere self-presentational artifact, as selflessness was uncorrelated to other-deception and associated with a broader and theoretically coherent pattern of relations with variables less readily reducible to social desirability, particularly self-loss and dissolution of self-boundaries. On the other hand, previous work has shown that self-deception is not reducible to a pure response bias and is also associated with several indicators of psychological adjustment and mental health [56,64–66]. Then, the positive association observed between selflessness and self-deception may reflect, at least in part, a positively valued self-view rather than only response bias, which would nuance the role of self-deception as a pure divergent validity indicator. In the present research, however, both interpretations cannot be distinguished given the split-questionnaire design. Discriminant validity with respect to social desirability, and especially self-deception, should therefore be considered only partially supported and requires further examination in future studies using jointly administered and more comprehensive criterion measures.

Regarding exploratory indicators, analyses depicted positive associations between selflessness and relationship quality, contrary to self-centeredness. Associations were broadly similar concerning relationship with nature: selflessness is associated with a positive relationship with nature (i.e., more concerns and behaviors) while self-centeredness is associated with a negative one. Taken together, these results were in line with the additional predictions of the SSHM, suggesting broader and daily effects of selflessness and self-centeredness than those observed on individual health only, in line with previous research [4].

Overall, findings were generally consistent with theoretical expectations regarding the validity of the scale, but should be interpreted cautiously. Because Study 3 relied on a split-questionnaire design, key variables such as social desirability and mental health were not assessed jointly in the same subsample, which precludes more decisive tests of their respective contributions. In addition, several external indicators were assessed using single-item visual analog scales or very brief composites. Although such measures are commonly used, they can provide useful general information and some have been related to fuller psychometric assessments in previous work [20,21,58–60], they remain relatively limited criterion variables for validating a newly developed multidimensional scale. Accordingly, the present results should be viewed as preliminary and theoretically coherent validity evidence.

## Cross-sample additional analyses

Following the analyses reported in Studies 1–3, additional analyses were conducted to further examine the robustness of the final scale structure. More specifically, these analyses aimed to determine whether the final measurement model operated equivalently across major sample and sociodemographic groups, and whether residual model misfit could partly reflect small cross-loadings not captured by the more restrictive confirmatory specification. To this end, we conducted both a supplementary comparison between confirmatory SEM and ESEM representations of the final structure, and measurement invariance analyses.

### Method

#### Participants and material.

The datasets from Studies 1, 2, and 3 were combined into a single database including the final 35-item version of the scale, as well as the sociodemographic variables used for grouping purposes. The combined sample comprised *N* = 2527 participants. The database included participants’ responses to the 35 scale items, together with information on sample membership (Study 1 *n* = 853, Study 2 *n* = 265, Study 3 *n* = 1409), gender (1769 females, 450 males), age, and self-reported social class (lower class *n* = 328, middle class *n* = 1634, upper class *n* = 290). To examine age-related invariance, age was recoded into two broad groups:18–29 years (*n* = 343) vs. 30 years and older (*n* = 1909). This grouping was guided by both the empirical distribution of the samples and by developmental literature identifying ages 18–29 as a distinct period of emerging adulthood [67].

#### Data analysis.

Complementary to analyses performed in Study 2, two SEM models were computed in this study to determine if cross-loadings contribute in part to a misfit in the whole sample. The first one was a confirmatory SEM model, while the second was a higher-order ESEM model, both were configured with the SSI-D hierarchical structure (i.e., seven first-order factors grouped into two second-order latent variables).

In addition, the measurement invariance of the final SSI-D structure was examined using multi-group structural equation modeling. Separate invariance analyses were conducted across (a) sample type, (b) gender, (c) broad age group, and (d) self-reported social class. Following classic recommendations, for each grouping variable, increasingly constrained models were estimated sequentially [68–70]. First, a configural invariance model was tested, in which the SSI-D structure was specified across groups, while all parameters were freely estimated. This model assesses whether the same general factorial structure holds across groups. Second, metric invariance was examined by constraining factor loadings to equality across groups. This step assesses whether the items relate to their respective latent dimensions with similar strength across groups. Third, scalar invariance was tested by additionally constraining intercepts to equality across groups. This step assesses whether group comparisons are affected by systematic response shifts at equivalent levels of the latent traits. Finally, strict invariance was examined by additionally constraining residual variances across groups.

All models were performed using the robust maximum likelihood method. The adequacy of the models to the data was evaluated with the following reference values: CFI > 0.90 (ideally 0.95), TLI > 0.90 (ideally 0.95), SRMR and RMSEA < 0.08 (ideally 0.05) [48,49]. Invariance was evaluated primarily on the basis of changes in fit indices across nested models, with particular attention to whether increasingly restrictive models resulted in only minor deterioration in fit. To compare models, reference fits were: Δ_CFI_ > 0.01, Δ_TLI_ > 0.01, Δ_RMSEA_ > 0.015, Δ_SRMR_ > 0.030 and Δ_χ²_ tests (*p* < .05) [50–52]. Greater emphasis was placed on approximate fit indices rather than on the chi-square statistic when interpreting model fit, given the sensitivity of this statistic to sample size and non-normality [53].

### Results

The confirmatory SEM model depicted acceptable fits: χ²(552) = 3558, *p* < .001; SRMR = 0.058; RMSEA = 0.046; 95% CI RMSEA [0.044, 0.047]; CFI = 0.936; TLI = 0.931; AIC = 296835; BIC = 297494. The higher-order ESEM model depicted satisfactory fits: χ²(363) = 1509, *p* < .001; SRMR = 0.013; RMSEA = 0.035; 95% CI RMSEA [0.034, 0.037]; CFI = 0.975; TLI = 0.959; AIC = 295164; BIC = 296926. Cross-loading values were systematically inferior to |β| = 0.20, indicating limited cross-load influences. The higher-order ESEM model fitted significantly better than the confirmatory SEM model according to reference criteria (Δ_*χ²*_ = 2049, *p* < .001; Δ_SRMR_ = 0.045; Δ_RMSEA_ = 0.011; Δ_CFI_ = 0.039; Δ_TLI_ = 0.028; Δ_AIC_ = 1671; Δ_BIC_ = 568). Cross-loadings significantly contribute to the misfit in confirmatory analyses, and contrary to Study 2, improvements are sufficient to reach strictly satisfactory thresholds.

#### Invariance analyses.

Fit indices for all invariance models are reported in Table 5.

**Table 5:** Fit indices for measurement invariance analyses across sample type, gender, age group, and self-reported social class.

Model	Chi-square	Degree of freedom	CFI	TLI	SRMR	RMSEA	95% CI RMSEA	AIC	BIC
Sample type									
Step 1	4961	1656	0.931	0.925	0.063	0.047	[0.045, 0.049]	295839	297817
Step 2	5102	1722	0.929	0.927	0.065	0.047	[0.045, 0.048]	295847	297440
Step 3	5459	1774	0.922	0.922	0.067	0.048	[0.047, 0.050]	296101	297391
Step 4	5905	1844	0.915	0.917	0.067	0.050	[0.048, 0.051]	296407	297288
Gender									
Step 1	3895	1104	0.934	0.929	0.062	0.046	[0.045, 0.048]	259806	261095
Step 2	3956	1137	0.934	0.931	0.062	0.046	[0.044, 0.048]	259801	260902
Step 3	4023	1163	0.933	0.931	0.062	0.046	[0.044, 0.047]	259816	260768
Step 4	4099	1198	0.932	0.933	0.062	0.045	[0.044, 0.047]	259822	260575
Age group									
Step 1	3854	1104	0.934	0.929	0.062	0.046	[0.044; 0.048]	263016	264308
Step 2	3905	1137	0.933	0.930	0.063	0.045	[0.044, 0.047]	263001	264105
Step 3	4134	1163	0.928	0.927	0.064	0.047	[0.045, 0.048]	263178	264133
Step 4	4476	1198	0.921	0.922	0.065	0.048	[0047, 0.050]	263450	264205
Social class									
Step 1	4502	1656	0.933	0.930	0.063	0.046	[0.044, 0.048]	263757	265696
Step 2	4600	1722	0.934	0.932	0.064	0.046	[0.044, 0.047]	263723	265284
Step 3	4801	1774	0.931	0.930	0.066	0.046	[0.044, 0.048]	263819	265084
Step 4	5001	1844	0.928	0.931	0.065	0.046	[0.044, 0.048]	263879	264743

Step 1: parameters were freely estimated (configural invariance), Step 2: constraining factor loadings to equality across groups (metric invariance), Step 3: additionally constraining intercepts to equality across groups (scalar invariance), Step 4: additionally constraining residual variances across groups (strict invariance).

The results of the multi-group analyses across the three samples supported configural invariance of the SSI-D structure. Imposing equality constraints on factor loadings and intercepts resulted in only negligible changes in fit (i.e., Δ_CFI_ < 0.01, Δ_TLI_ < 0.01, Δ_RMSEA_ < 0.015, Δ_SRMR_ < 0.030), supporting both metric and scalar invariance across samples. When residual variances were further constrained, CFI indices showed a significant decline (Δ_CFI_ = 0.016 compared to the configural model and Δ_CFI_ = 0.014 compared to the metric model), suggesting that strict invariance was less supported than the lower levels of invariance. Overall, these findings indicate that the scale displayed a broadly stable measurement structure across the three samples, with acceptable evidence at the configural, metric, and scalar levels.

The analyses across gender provided support for measurement invariance. The configural model showed acceptable fit, and fit indices did not deteriorate when equality constraints were imposed on loadings, intercepts, and residual variances (i.e., Δ_CFI_ < 0.01, Δ_TLI_ < 0.01, Δ_RMSEA_ < 0.015, Δ_SRMR_ < 0.030). Taken together, the results support configural, metric, scalar, and strict invariance across gender.

The analyses comparing participants aged 18–29 years and those aged 30 years and older also broadly supported invariance of the SSI-D structure. Transitions from the configural to the metric model and from the metric to the scalar model were associated with no change in fit (i.e., Δ_CFI_ < 0.01, Δ_TLI_ < 0.01, Δ_RMSEA_ < 0.015, Δ_SRMR_ < 0.030). The strict invariance model showed significant decline in CFI (Δ_CFI_ = 0.013 compared to the configural model and Δ_CFI_ = 0.012 compared to the metric model), suggesting a more cautious interpretation at that level. Overall, the results support configural, metric, and scalar invariance across age groups.

Additional analyses conducted across self-reported social class categories depicted an acceptable SSI-D structure reproduced across groups. The imposition of equality constraints resulted in no meaningful deterioration in model fit (i.e., Δ_CFI_ < 0.01, Δ_TLI_ < 0.01, Δ_RMSEA_ < 0.015, Δ_SRMR_ < 0.030). These findings suggest that the scale also operates comparably across broad levels of self-perceived social position.

### Discussion

The additional analyses provided further evidence regarding the robustness of the final scale structure. First, the comparison between the confirmatory SEM and ESEM representations were informative with respect to the residual misfit observed in the confirmatory model. Although the SEM solution showed an acceptable fit, the ESEM solution yielded a satisfactory and significantly better fit. Interestingly, no cross-loading reached the threshold of |β| > 0.20. This pattern suggests that part of the remaining misfit in the confirmatory specification is likely attributable to overall small cross-loadings that are fixed to zero in the more restrictive SEM framework. In other words, the data indicate that the item structure is somewhat more complex than a strictly simple-structure confirmatory model would assume.

Importantly, this finding does not undermine the substantive relevance of the SEM solution. Rather, it suggests that the confirmatory model still provides a parsimonious and theoretically coherent representation of the data, while the ESEM results highlight a significant degree of item-level complexity. This pattern is not unexpected in the present case, as several items assigned to distinct dimensions in the SSI-D were originally drawn from the same source measures, and the dimensions themselves are conceptually related. Under such conditions, the presence of modest cross-loadings is theoretically plausible and does not necessarily contradict the broader hierarchical organization retained in the confirmatory analyses.

Second, the multi-group invariance analyses indicated that the SSI-D structure operated in a broadly comparable manner across samples and major sociodemographic groups. The scale structure was consistently supported across sample type, gender, age group, and self-reported social class, with acceptable evidence for configural, metric, and scalar invariance. Evidence for strict invariance was somewhat less consistent, especially across sample type and age group.

Overall, these findings converge in supporting the validity and the generalizability of the scale. Thus, the final model appears both theoretically meaningful and psychometrically acceptable. At the same time, some caution is warranted. Because the compared groups were not fully balanced in size, the fit indices may have been influenced more strongly by the larger groups, even though the smaller groups were sufficiently large for model estimation. Replication in more evenly balanced samples would therefore be valuable. In addition, all participants were drawn from the same broader cultural context (i.e., French samples), indicating that the present findings support the stability of the scale across samples and sociodemographic groups within that context, but do not by themselves support broader cross-cultural generalization.

## General discussion

The aim of this research was to develop a day-level version of the Selflessness/Self-centeredness Inventory to assess forms of psychological functioning at a more restricted level and closer to experience than overall dispositions. Selflessness and self-centeredness were presented as two distinct functionings that anybody could experience in different situations [2,3]. To develop the scale, daily experiences of selflessness and self-centeredness have been defined by distinct components reflecting the observed trait structure [4]. To evaluate these dimensions, the SSI-D was developed on the basis of three studies involving a total of 2,527 participants. The relationships between the scale and various categories of correlates were analyzed, including self-loss, dissolution of self-boundary dimensions, and mental health. In addition, indicators of relationship quality and relationship with nature were included to explore broader effects of selflessness and self-centeredness. The results obtained from these studies were consistent with previous literature and broadly supported reliability and validity of the scale.

The results of the factor analyses performed across studies provide initial structural evidence that day-level selflessness and self-centeredness can be represented as two higher-ordered variables characterized by distinct factors. At the day-level, the SSI-D showed a structure broadly consistent with the trait-version [4]: four dimensions representing selflessness (i.e., connection with humanity, connection with nature, decentering, and compassion) and three dimensions representing self-centeredness (i.e., self-focus, self-importance, and hedonic process). Selflessness’s structure matches with its definition as the reduced perception of being an entity separated from its environment, leading to a kind of undifferentiation between self and the environment [2–4]. Self-centeredness structure also matches with its definition, characterized by a strong distinction between the self and its environment associated with a high self-attribution of mental processes. Moreover, motivational aspects of each functioning— respectively, the principle of harmony for selflessness and the hedonic principle for self-centeredness—appear to be captured by the proposed structure. Consequently, the scale presents a psychometrically acceptable and theoretically coherent structure for reflecting day-level self-functioning components, consistent with its trait equivalent [4], although the only acceptable fit observed in Study 2 indicates that further independent replication remains needed. To complete, the factorial structure of the scale is in line with theoretical expectations regarding the dissociation and qualitative distinction between the two psychological functionings [2]. At the same time, first-order models and models including the two broader latent variables were repeatedly found to be empirically equivalent in Studies 1 and 2. The hierarchical representation was therefore retained primarily on theoretical and parsimonious grounds, in line with the SSHM, rather than because it was unequivocally superior statistically. This result is itself informative, as it suggests that the SSHM can be studied meaningfully both at the level of the two broad functionings and at the level of their specific components. In addition, correlation between selflessness and self-centeredness varied across studies but remained weak overall, ranging from a small negative association to a non-significant relation. This pattern is consistent with previous trait literature [4,5] and with expectations that selflessness and self-centeredness, at the day-level, are neither totally independent or two opposite poles on a linear continuum. Each form of functioning possesses its own continuum of activity, and it might be possible to experience both during the same short period of time. Given the differences in sample composition across studies, it is also possible that the relation between selflessness and self-centeredness may vary as a function of demographic, contextual, or cultural characteristics, a possibility that future research should examine more directly.

Complementary analyses also help qualify and clarify these structural conclusions. In Study 2, and similarly in cross-sample analyses, ESEM models provided somewhat better fit than the corresponding SEM models, indicating that allowing cross-loadings captured part of the observed misfit. This result is not unexpected, as several SSI-D items originated from common source measures and may therefore retain some meaningful overlap across neighboring dimensions. Such cross-loadings can thus be understood as theoretically interpretable rather than as evidence against the retained structure. Finally, the invariance analyses provided additional support for the generalizability of the structure across the present samples. Configural, metric, and scalar invariance were broadly supported across samples, gender, age groups, and self-reported social class. Strict invariance was more mixed, calling for some caution regarding full residual equivalence. Taken together, these results support a meaningful degree of generalizability within the present data, while also indicating that future work should test the SSI-D in more demographically balanced samples and in other cultural contexts. Indeed, the present samples were composed of French participants, and the findings should therefore be interpreted primarily within this cultural context. Although the SSHM is intended to describe general modes of psychological functioning, their expression, salience, or social value may vary across cultural settings [2]. Testing the SSI-D in other countries and cultural groups would therefore be important to clarify the cross-cultural validity and transferability of the present findings.

Assessment of the divergent validity of the selflessness subscale indicated no association between the scale and other-deception, gender, educational level, and social class. These findings are broadly consistent with previous studies that used such indicators to document divergent validity [10,14,71]. However, as discussed in Study 3, the significant correlation between selflessness and self-deception calls for cautious interpretation and tempers the evidence for discriminant validity, which should therefore be considered only partially supported at this stage. It raises the possibility that selflessness may overlap with self-enhancing or idealized self-perceptions. This association may also reflect a positively valued self-view rather than a simple generalized faking-good response pattern [56,64–66], consistent with the absence of significant associations with other-deception. Still, the present data do not allow these interpretations to be disentangled directly, notably because mental health and social desirability were not assessed within the same sub-questionnaire in Study 3. Future research should therefore administer these measures jointly to clarify whether the association with self-deception reflects adjustment-related variance, self-enhancement, or both. Concerning convergent validity, selflessness is linked to some indicators of self-loss and dissolution of self-boundaries. Especially, correlations with unity with all things, boundary diminishment with others, and boundary diminishment with objects, as well as with loss of space and time, and the salience of body boundaries are consistent with the definition of selflessness as characterized by a sense of connection beyond the self. That illustrates the close link between selflessness and relational transcendence [2]. Also, positive correlations between selflessness and mindfulness dimensions are in line with previous studies and definitions [2,3,6,17]. Finally, correlations with mental health indicators were broadly in the expected direction, with selflessness relating positively to positive mental health and especially authentic-durable happiness [2,6,20,21]. Given that several criterion variables were assessed using single-item VAS or very brief indicators, these associations should be interpreted as theoretically coherent, though still preliminary, validity evidence for the scale, consistent with their equivalent at the trait-level [4,5,16].

Regarding the validity of the self-centeredness subscale, as for selflessness, no associations were found with gender, educational level, social class, and other-deception, consistent with divergent validity [10,14,71]. However, as discussed above for selflessness, significant correlations involving self-deception should be interpreted cautiously and need further research to clarify the processes underlying this association. Hence, evidence concerning divergent validity are only partially supported and require further validation studies. Regarding convergent validity, significant correlations between self-centeredness and self-loss and dissolution of self-boundaries were inconsistent with predictions, but since they became insignificant when controlling positive and negative affect (reflecting a confounding effect), such a finding is meaningful. Indeed, as all these dimensions have implications for well-being [3,10,26]. Then, overall independence between self-centeredness and self-loss and dissolution of self-boundaries are congruent with predictions, as self-centeredness is characterized by a unique and stable self separated from its surroundings [3]. Two correlations remain slightly significant, but can be explained by the cultural interpretation given to the experience and by the presence of absorption in self-loss experiences [10,11]. With the same reasoning, independence between self-centeredness and curiosity dimension of mindfulness is consistent with the absence of self-alteration in self-centeredness, while the negative correlation with the decentering dimension illustrates the excessive focus on thoughts, feelings, and sensations (i.e., experiential fusion), a marker of self-centered activity [3]. Finally, when focusing on mental health correlates, associations are opposed to those with selflessness as predicted, and consistent with what is observed at the trait-level [4,5,9,16]. Taken together, these findings are consistent with expectations, but should still be considered preliminary evidence for the convergent validity of the self-centeredness subscale, in line with the methodological limits of Study 3.

Looking ahead more broadly, in addition to the results on mental health, results concerning relationship quality and the relationship with nature open interesting perspectives. Indeed, selflessness is associated with a higher perception of relationship quality (including lower affective conflicts). Selflessness is also associated with more ecological concerns and intentions to behave in an eco-friendly way, without any link with ecoanxiety. That can be explained by the presence of compassion and connection with both others and nature in the daily experience of selflessness (depicting the inclusion of the environment in the self, and the principle of harmony), leading to a better appreciation of others and nature, and decreasing related negative affects (e.g., frustration, anger). This is consistent with hypotheses and previous literature on the link between self-transcendence and pro-environmental outcomes [4,72–74], as well as specific effects of both connectedness with humanity and with nature [27,75]. Taken together, these relationships depict day-level selflessness as a positive functioning not only for mental health, but also for relationship quality, and the relationship with nature. Conversely, self-centeredness is associated with worst relationship quality (including higher affective conflicts) as well as with more ecoanxiety but less ecological concerns and less intentions to behave in an eco-friendly way. In other terms, day-level self-centeredness seems to decrease the appreciation of others and to promote negative affectivity toward them. Also, more ecoanxiety, less consciousness of the impacts of ecological issues and less intentions to act in an eco-friendly way are reported on a day high in self-centeredness. These phenomena are particularly illustrative of the excessive focus on the self and its separation from its social and physical environment in self-centeredness [3,4]. Taken from a global perspective, these results depict self-centeredness as a negative functioning with relation to mental health, relationship quality, and relationship with nature. Hence, the SSHM framework offers two predictors of various indicators of health: direct indicators of mental health, indirect indicators of interpersonal health through relationship quality, and indirect indicators of environmental health, especially through ecological behavior considered as health behaviors to nature. Importantly, these associations were observed at the day-level rather than as broad life tendencies. In other words, days marked by higher (or lower) selflessness or self-centeredness were associated with different levels of mental health, interpersonal functioning, and environment-related outcomes.

Concerning additional future research, an important step would be to examine the functioning of the SSI-D across repeated assessments from one day to another, in order to better capture the temporal dynamics of selflessness and self-centeredness day-levels. Such designs would make it possible to determine more precisely how these modes of functioning fluctuate over time, and how they relate to day-to-day contextual factors and variations in mental health. Interestingly, given the day-based perspective of the SSHM, the SSI-D may be well-suited for intensive longitudinal monitoring in high-demand contexts. In occupational health, repeated SSI-D assessments could help track daily changes in self-related functioning in high-stress populations, and model within-person links with exhaustion and burnout indicators. Indeed, recent work mapping research trends on medical resident burnout and physical activity illustrates the growing attention to the burnout-activity interface and the need for designs clarifying mechanisms over time [76]. In parallel, sport and high-performance research commonly rely on mood balance indices as load-sensitive snapshots, and training constraints can be manipulated to shift mood balance alongside technical performance [77]. The SSI-D could complement such approaches by capturing self-related mechanisms that may amplify or dampen affective fluctuations associated with daily demands and external loads.

With a more methodological perspective, additional studies could also incorporate basic physiological covariates (e.g., sleep duration/quality and time-of-day) to better disentangle psychobiological sources of day-to-day variation in SSI-D scores and related outcomes [25,29]. Indeed, as mentioned in the Methods section in Study 1, daily reports are partially shaped by biological rhythms, what should be investigated. This consideration may be particularly relevant for selflessness-related phenomenological facets such as timelessness, which may partly reflect psychobiological states (e.g., sleep) and their interaction with interpretative frameworks [78]. Another important protocol in terms of validity and reliability would be to assess the relations between the current scale and its trait version (i.e., the Selflessness/Self-centeredness Inventory – Trait) [4]. Such a study would allow us to better understand the links between overall dispositions and more restricted constructs, as both are seen to influence themselves. These empirical linkages would also concern the cognitive processes underlying selflessness, such as impermanence and third-person phenomenology [2,19].

Finally, in line with previous studies on the state-level [20,21], an important perspective might be the use of the experience-sampling method (ESM) to test even more refined, momentary versions of the SSI-D, capturing “in-the-moment” states with minimal retrospective reconstruction. Indeed, although the use of “today” as a reference frame assesses selflessness and self-centeredness over a shorter and more delimited period than trait-level measures, it does not correspond to a true momentary assessment in the sense of ESM protocols. Such daily reports still involve brief retrospective reconstruction and may therefore be affected by memory and appraisal biases [25]. Relatedly, ESM protocols could then be used to assess prospective effects of selflessness and self-centeredness (either from one day to the next with the current scale, or from one moment to another with an “in-the-moment” version). Since the present research was cross-sectional and relied only on subjective reports (giving no direct observation of immediate levels and temporal variability), longitudinal and experience-sampling designs would therefore make it possible to assess both immediate levels of variables and temporal fluctuations of them, bringing complementary findings to the current research. Beyond replicating effects concerning selflessness, such studies would also inform about the effects of immediate self-centeredness, which has not yet been investigated at the state-level [4,20,21].

## Conclusion

To conclude, the present article developed and provided initial validation evidence for the Selflessness/Self-centeredness Inventory – Day-level (SSI-D), a psychometric instrument for assessing daily levels of selflessness and self-centeredness from the SSHM framework. Across three studies, the seven-factor structure (four selflessness components and three self-centeredness components) was broadly consistent with the trait taxonomy and showed acceptable reliability and invariance indicators. While nuanced validity evidence called for further considerations, especially regarding social desirability, findings indicated that day-level selflessness relates to greater happiness, perceived relational quality, and pro-environmental support, whereas day-level self-centeredness correlates to negative mental health, reduced relationship quality, and ecoanxiety. Thus, that research provides a useful tool for future day-level protocols, complementary to overall dispositional research.

## Supporting information

S1 File Selflessness/Self-centeredness Inventory – Day-level (SSI-D), English Version. (PDF)
