# Supplementary material for: From traits to daily experiences: An adaptation of the Selflessness/Self-centeredness Inventory to day-level assessment
Source: PLoS One. 2026 Jun 23;21(6):e0351154. doi: 10.1371/journal.pone.0351154 (PMC13289953; doi:10.1371/journal.pone.0351154)
Supplement: S1 File — (PDF) [file pone.0351154.s001.pdf]

Selflessness/Self-centeredness Inventory – Day level (SSI-D)

English Version

(David, Pellerin, & Dambrun)

Note. Items should be presented in a random order.

We are interested in your current experiences. Below is a list of things people sometimes experience. Using the scale, please indicate for each statement how much you experience this today.

1	2	3	4	5	6	7
Not at all	Very weakly	Quite weakly	Moderately	Quite strongly	Very strongly	Totally

Today, I think of human beings as part of a single group.	1 2 3 4 5 6 7
Today, I think of human beings as part of one large family.	1 2 3 4 5 6 7
Today, I have a sense of "we-ness" with all human beings.	1 2 3 4 5 6 7
Today, I have a feeling of unity with others.	1 2 3 4 5 6 7
Today, I share the same identity with all human beings.	1 2 3 4 5 6 7
Today, I feel a strong connection between myself and the Earth.	1 2 3 4 5 6 7
Today, I feel a strong connection between myself and a tree.	1 2 3 4 5 6 7
Today, I feel a strong connection between myself and an eagle soaring in the sky.	1 2 3 4 5 6 7
Today, I feel at one with the universe.	1 2 3 4 5 6 7
Today, I experience myself as extending into everything else.	1 2 3 4 5 6 7
Today, I want to support others.	1 2 3 4 5 6 7
Today, I want to help people around me and care for them.	1 2 3 4 5 6 7
Today, I want to be there for others in times of difficulty.	1 2 3 4 5 6 7
Today, I want to make a positive difference in someone else's life.	1 2 3 4 5 6 7
Today, I want to be loyal and to devote myself to people close to me.	1 2 3 4 5 6 7
Today, I am aware of unpleasant thoughts or feelings without immediately reacting to them.	1 2 3 4 5 6 7
Today, I am able to notice distressing thoughts or feelings without reacting.	1 2 3 4 5 6 7

Today, I watch my thoughts and emotions come and go like clouds.	1 2 3 4 5 6 7
Today, I am able to watch my thoughts and feelings like someone watching a movie.	1 2 3 4 5 6 7
Today, I am open to observing unpleasant thoughts and feelings without interfering with them.	1 2 3 4 5 6 7
Today, I have distressing thoughts spinning on a loop in my head.	1 2 3 4 5 6 7
Today, I am affected by negative thoughts and emotions.	1 2 3 4 5 6 7
Today, I ruminate or dwell over things that happen to me.	1 2 3 4 5 6 7
Today, I keep going back to what happened during a past argument or disagreement.	1 2 3 4 5 6 7
Today, I rehash in my mind recent things I've said or done.	1 2 3 4 5 6 7
Today, I want to show my abilities, I want people to admire what I do.	1 2 3 4 5 6 7
Today, I want others to admire me.	1 2 3 4 5 6 7
Today, I want to be successful, I would like to impress other people.	1 2 3 4 5 6 7
Today, I want to get others to recognize or acknowledge my positive qualities.	1 2 3 4 5 6 7
Today, I want others to pay attention to me.	1 2 3 4 5 6 7
Today, I want to be rich, to have a lot of money and expensive things.	1 2 3 4 5 6 7
Today, I like to have a lot of luxury in my life.	1 2 3 4 5 6 7
Today, I imagine my life better if I owned some things I don't have.	1 2 3 4 5 6 7
Today, I think my life would be better if I owned certain things I don't have.	1 2 3 4 5 6 7
Today, I admire people who own expensive homes, cars, and clothes.	1 2 3 4 5 6 7
